# Haplotype-resolved assemblies and variant benchmark of a Chinese Quartet

**DOI:** 10.1186/s13059-023-03116-3

**Published:** 2023-12-04

**Authors:** Peng Jia, Lianhua Dong, Xiaofei Yang, Bo Wang, Stephen J. Bush, Tingjie Wang, Jiadong Lin, Songbo Wang, Xixi Zhao, Tun Xu, Yizhuo Che, Ningxin Dang, Luyao Ren, Yujing Zhang, Xia Wang, Fan Liang, Yang Wang, Jue Ruan, Han Xia, Yuanting Zheng, Leming Shi, Yi Lv, Jing Wang, Kai Ye

**Affiliations:** 1 National Local Joint Engineering Research Center for Precision Surgery & Regenerative Medicine, Center for Mathematical Medical, The First Affiliated Hospital of Xi’an Jiaotong University, Xi’an, 710061 China; 2 School of Automation Science and Engineering, Faculty of Electronic and Information Engineering, Xi’an Jiaotong University, Xi’an, 710049 China; 3 MOE Key Lab for Intelligent Networks & Networks Security, Faculty of Electronic and Information Engineering, Xi’an Jiaotong University, Xi’an, 710049 China; 4 National Institute of Metrology, Beijing, 100029 China; 5 School of Computer Science and Technology, Faculty of Electronic and Information Engineering, Xi’an Jiaotong University, Xi’an, 710049 China; 6 Genome Institute, The First Affiliated Hospital of Xi’an Jiaotong University, Xi’an, 710061 China; 7 State Key Laboratory of Genetic Engineering, Human Phenome Institute, School of Life Sciences and Shanghai Cancer Center, Fudan University, Shanghai, 200438 China; 8 GrandOmics Biosciences, Beijing, 100089 China; 9 Shenzhen Branch, Guangdong Laboratory of Lingnan Modern Agriculture, Genome Analysis Laboratory of the Ministry of Agriculture and Rural Affairs, Agricultural Genomics Institute at Shenzhen, Chinese Academy of Agricultural Sciences, Shenzhen, 518120 China; 10 School of Life Science and Technology, Xi’an Jiaotong University, Xi’an 710049, China; 11 Faculty of Science, Leiden University, Leiden, 2311EZ The Netherlands

## Abstract

**Background:**

Recent state-of-the-art sequencing technologies enable the investigation of challenging regions in the human genome and expand the scope of variant benchmarking datasets. Herein, we sequence a Chinese Quartet, comprising two monozygotic twin daughters and their biological parents, using four short and long sequencing platforms (Illumina, BGI, PacBio, and Oxford Nanopore Technology).

**Results:**

The long reads from the monozygotic twin daughters are phased into paternal and maternal haplotypes using the parent–child genetic map and for each haplotype. We also use long reads to generate haplotype-resolved whole-genome assemblies with completeness and continuity exceeding that of GRCh38. Using this Quartet, we comprehensively catalogue the human variant landscape, generating a dataset of 3,962,453 SNVs, 886,648 indels (< 50 bp), 9726 large deletions (≥ 50 bp), 15,600 large insertions (≥ 50 bp), 40 inversions, 31 complex structural variants, and 68 de novo mutations which are shared between the monozygotic twin daughters. Variants underrepresented in previous benchmarks owing to their complexity—including those located at long repeat regions, complex structural variants, and de novo mutations—are systematically examined in this study.

**Conclusions:**

In summary, this study provides high-quality haplotype-resolved assemblies and a comprehensive set of benchmarking resources for two Chinese monozygotic twin samples which, relative to existing benchmarks, offers expanded genomic coverage and insight into complex variant categories.

**Supplementary Information:**

The online version contains supplementary material available at 10.1186/s13059-023-03116-3.

## Background

Human genomic variants, including single nucleotide variants (SNVs), small insertions/deletions (indels), and structural variants (SVs), have been extensively characterized and contributed to many diseases [1–4]. Authoritative and comprehensive variant benchmarks are therefore crucial for precisely understanding genetic variation in clinical samples. During the past decades, many consortiums such as Genome in a Bottle [5–10] (GIAB), Sequencing Quality Control [11–19], and Illumina Platinum Genomes [20] have established many variant benchmarks and genomic reference materials [19, 21, 22]. These resources help the community evaluate their variant detection strategies. Nevertheless, the majority of these studies characterize simple variant types and regions, with complex events and regions (such as those which are repetitive) generally underrepresented. Benchmarks for complex structural variants and de novo mutations are similarly underrepresented and of particular value, given the critical role they play in human health and disease [23–27].

Advanced sequencing technologies [28–30], including PacBio HiFi and Oxford Nanopore ultra-long reads, were recently leveraged to assemble a complete hydatidiform mole from telomere to telomere [31] (CHM13-T2T), making it possible to resolve the sequences of many medically-related genes and regions excluded by previous benchmarking resources [32]. Recently, several studies have also demonstrated that high-quality haplotype-resolved assemblies (HRAs) can detect many more variants than previous read-alignment-based strategies [33–38] and resolve those in more complex regions, such as simple repeats (SRs), short tandem repeats (STRs), variable number tandem repeats (VNTRs), and segmental duplications (SDs). Alongside the growing prevalence of longer reads and higher-quality assemblies, novel computational methods such as Sniffles [39], cuteSV [40], and SVision [23] have increasingly revealed complex SVs in the human genome.

Herein, we present the results of the Chinese Quartet Project, which constructs haplotype-resolved assemblies and variant benchmarking resources for the Chinese Han population by sequencing a “Chinese Quartet” of two monozygotic twin daughters (LCL5 and LCL6) and their biological parents (LCL7 and LCL8). The DNA of the four samples has been approved as Certified Reference Materials for whole genome-variant assessment by the State Administration for Market Regulation in China [41]. We sequenced four related individuals because with single samples (or even a mother/father/child trio) random erroneous or variation may be introduced—and remain uncorrected—by contamination in cell line culture and transportation [42]. We sequenced each of four samples using four sequencing technologies (Illumina, BGI, PacBio, and ONT) and assembled whole-genome and haplotype-resolved genomes for the monozygotic twins and collapsed genomes for parents. We demonstrated that the two haplotypes of the twins achieved high performance in terms of accuracy, continuity, and completeness, typically exceeding that of GRCh38, and used the quartet resources to generated a comprehensive catalogue of human variants, including underrepresented categories of simple germline variants, complex structural variants, de novo mutations, and putative somatic mutations.

## Results

### Sample processing and sequencing

To obtain high-quality genome assemblies for the Chinese Quartet, we generated approx. 50x HiFi (read length N50 = 13–14 kb), approx. 100x regular ONT (read length N50 = 20–25 kb) reads for each of four samples, and in addition approx. 30x ultra-long ONT (read length N50 = 77 kb) reads for one twin sample, LCL5 (Additional file 1: Table S1). To establish a robust variant benchmark for the twin daughters, we used approx. 160x Illumina NovaSeq (150 bp paired-end) and approx. 100x BGI (100 bp paired-end) reads alongside previously described a variety of long reads (Additional file 1: Table S1 and Additional file 2: Fig. S1).

### Haplotype-resolved genome assembly

Since monozygotic twins are generally considered genetically identical with limited somatic substitutions [43], we merged the reads from these two samples to generate a high-quality haplotype-resolved genome. We phased HiFi, regular ONT, and ultra-long ONT reads of the monozygotic twins into paternal (CQ-P) and maternal (CQ-M) haplotypes and assembled each haplotype using a hybrid assembly strategy (Additional file 2: Fig. S2). First, 3,249,650 high-quality SNVs and 404,882 indels were obtained from a previous study [11] and phased according to parent–child information and each child’s HiFi reads [44]. Next, HiFi, regular ONT, and ultra-long ONT reads from the two twin daughters were separated into two haplotypes using the phased variants [44]. Overall, we phased 76.2% of the HiFi reads, 65.0% of the regular ONT reads, and 72.8% of the ultra-long ONT reads, with all unmapped or unphased reads assigned to the two haplotypes randomly (Additional file 1: Table S2). For each haplotype of the two twin daughters, we obtained coverage of around 53 × HiFi, 95 × regular ONT, and 14 × ultra-long ONT reads (Additional file 1: Table S2). We independently assembled the ONT reads using shasta [45] and flye [46] and the HiFi reads using hifiasm, hicanu [47], and flye [46], producing five haplotype-resolved assemblies, each representing the pairwise combination of a different sequencing technology and assembler (Additional file 1: Table S3). After that, the hifiasm contigs were scaffolded using ragtag [48] and the other four assemblies were used to fill the gaps in the hifiasm scaffolds (see the “Methods” section and Additional file 3). Finally, the two haplotypes of twin daughters were further polished with phased HiFi reads [49].

**Fig. 1:**
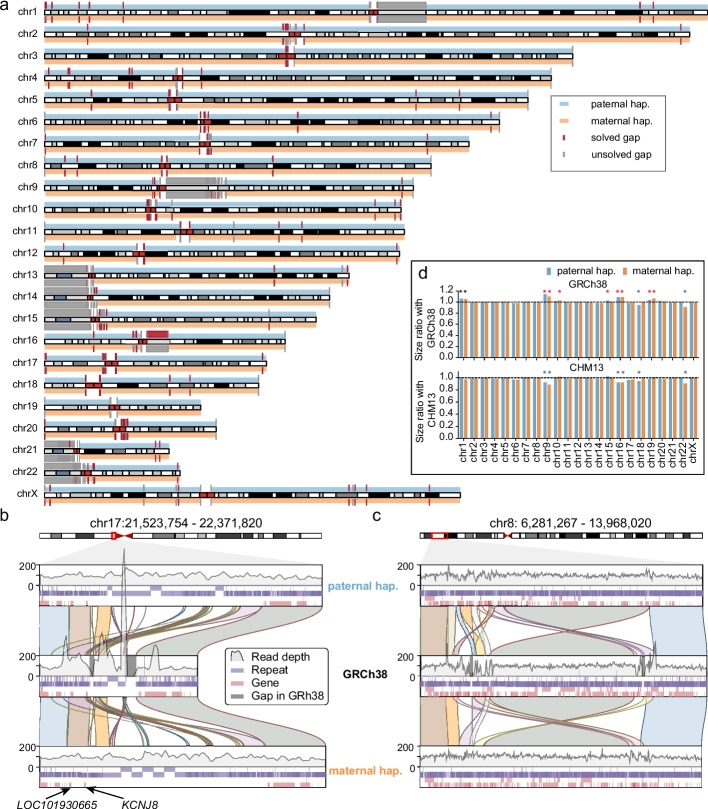
An overview of the Chinese Quartet assemblies. **a** Idiogram depicts the alignments between the GRCh38 (gray rectangles) and two Chinese Quartet haplotypes (blue rectangles for CQ-P and orange for CQ-M). The red rectangles represent the GRCh38 gaps filled by Chinese Quartet assemblies, while the gray rectangles refer to unresolved gaps. **b**, **c** Examples of gaps resolved by Chinese Quartet assemblies. The top and bottom channels represent the paternal and maternal haplotypes, respectively. The middle channel represents the GRCh38. The depths of HiFi reads on three genomes are shown with gray lines. The repeat regions and genes are labeled with purple and pink rectangles, and the gaps in GRCh38 are labeled with gray rectangles. **d** The bar plots show the percentage size of Chinese Quartet assembled chromosomes relative to CHM13-T2T (top) and GRCh38 chromosomes (bottom), without including Ns. The chromosome with more than 3% difference in length is labeled with a star

The final two haplotypes comprised 297 contigs for CQ-P and 276 contigs for CQ-M, with both having a length of 3.05 Gb (Table 1). The contig N50 values of two haplotypes were each approx. 133 M, about twofold that of GRCh38.p13, suggesting a high contiguity of the phased assemblies compared to previous reports [50–54] (Table 1 and Additional file 1: Table S4). Notably, seven and nine chromosomes from the paternal and maternal haplotypes, respectively, were gap-free from telomere to telomere. Similarly, 20 chromosome arms in CQ-P and 18 chromosome arms in CQ-M were gap-free from telomere to centromere (Additional file 1: Table S5 and Additional file 2: Figs. S3, S4). Furthermore, CQ-P and CQ-M closed 236 and 251 gaps in GRCh38, respectively (Fig. 1a and Additional file 2: Fig. S5). For example, gaps in GRCh38 near the centromere of chromosome 17 were filled by both CQ-P and CQ-M haplotypes (Fig. 1b and Additional file 2: Figs. S6, S7). In addition, a 4 M polymorphic inversion by CHM13-T2T [55] at chromosome 8p23.1 was also resolved in both haplotypes (Fig. 1c and Additional file 2: Figs. S6, S8).

**Table 1:** Summary statistics and comparison of the haplotype-resolved Chinese Quartet assemblies to other assemblies

Sample	Haplotype	Genome length (Gb)	No. of contigs	Contig N50 (Mb)	Completeness (BUSCO)	QV	Switch error
Chinese Quartet	Paternal	3.05	279	132.84	95.7%	50–58	0.050%
	Maternal	3.05	276	132.84	95.7%	52–59	0.048%
HJ [50]	Paternal	3.07	1330	28.15	94.9%	52–59	0.815%
	Maternal	2.91	896	25.90	93.5%	54–58	0.813%
NA12878 [51]	Hap1	2.88	4363	18.3	95.5%	51–60	0.449%
	Hap2	2.88	4449	21.9	95.4%	51–60	0.435%
HG00733 [51]	Hap1	2.92	3728	23.7	94.9%	50–59	0.169%
	Hap2	2.92	3795	25.9	95.1%	51–59	0.171%
HG002 [56]	Paternal	2.96	631	84.93	93.7%	NA	NA
	Maternal	3.06	464	62.88	95.9%	NA	NA
HPRC [36]^a^	Paternal	3.00	439	40.36	95.0%	NA	NA
	Maternal	3.04	378	40.90	95.9%	NA	NA
YH2.0 [54]	Collapsed	2.91	361,157	0.02	94.2%	NA	NA
HX1 [52]	Collapsed	2.93	5845	8.33	94.0%	NA	NA
NH1.0 [53]	Collapsed	2.89	11,019	3.6	94.6%	NA	NA
GRCh38.p13^b^	Collapsed	3.21	685	56.41	94.7%	NA	NA
CHM13-T2T(v2.0) [31]	/	3.12	25	150.6	96.0	NA	NA

*NA* not available

^a^The average performances of HPRC project were calculated according to 47 assemblies

^b^GRCh38 without the alternative sequences

We demonstrated that ten chromosomes (5 paternal and 5 maternal) of our assemblies had more than a 3% increase in length compared with GRCh38, while six chromosomes (3 paternal and 3 maternal) had a 3% decrease in length compared to CHM13-T2T (Fig. 1d). To further assess the completeness of CQ-P and CQ-M, we aligned both haplotypes against GRCh38 and observed that CQ-P and CQ-M covered 97.59% and 97.55% of the GRCh38 genome, respectively (Additional file 1: Table S6). Completeness evaluation by BUSCO [57] (v5.1.3) showed that our phased genomes resolved 95.7% of the complete genes from the mammalia_odb10 library, which was higher than three previous Chinese assemblies [52–54] and comparable to the recent reports of HJ [50] and HPRC [36, 56] (Table 1 and Additional file 1: Table S7).

To comprehensively characterize the assemblies, we annotated genes and novel sequences on both haplotypes (Additional file 2: Fig. S9). We found 8172 (8.4 Mb) and 8175 (8.8 M) novel sequences in CQ-M and CQ-P, respectively, when compared to GRCh38. The N50 of novel sequences in CQ-M and CQ-P were 16.2 kb and 13.1 kb, respectively. We also found that only 0.87% (143) and 0.99% (162) of novel sequences could be mapped to the CHM13-T2T [31] (v2.0) and HPRC [36] genomes, respectively. Most novel sequences were located in centromeric and acrocentric regions (Additional file 2: Fig. S10). To annotate our genomes, we converted the gene coordinates of GRCh38.p13 (chr1-chr22, and chrX) to CQ-P and CQ-M using Liftoff [58] (v1.6.1), of which 96.69% (19,221/19878) and 96.62% (19,208/19878) of protein-coding genes were successfully converted (Additional file 1: Table S7). To annotate genes within novel sequences, we masked repeats and annotated the protein-coding genes by Augustus [59] (v3.4.0). Finally, we obtained 45 and 58 novel genes in CQ-P and CQ-M, respectively (Additional file 1: Table S8), of which four were successfully mapped to the CHM13-T2T [31] (v2.0) and HPRC [36] genomes.

### Construction of variant benchmarking set

Since each sequencing technology and variant detection pipeline had its own advantages, we utilized short reads, long reads, and haplotype-resolved assemblies to call variants for the monozygotic twins (Additional file 2: Figs. S11, S12). To eliminate false positives caused by random errors, we implemented a rigorous filter for germline variants based on Mendelian inheritance laws (Additional file 3). Specifically, we only kept those variants supported by both twins and at least one parent in the benchmark. For variants which did not adhere to Mendelian inheritance laws or were not present in either parent, we considered them as de novo mutations if shared by both twins, and as putative somatic mutations if supported by only one daughter.

#### SNV and indel benchmark construction

Illumina-based SNV and indel calls were downloaded from a previous study [11]. HiFi calls were generated by the minimap2-DeepVariant pipeline [60, 61]. Both the Illumina and HiFi calls were filtered by read depth, allele frequency, and the Mendelian rule (Additional file 3). Meanwhile, three haplotype-resolved assemblies (generated with HiFi reads) were used for variant discovery by PAV [33]. Only variants supported by all three assemblies were included in the HRA callset (see the “Methods” section and Additional file 2: Fig. S11).

**Fig. 2:**
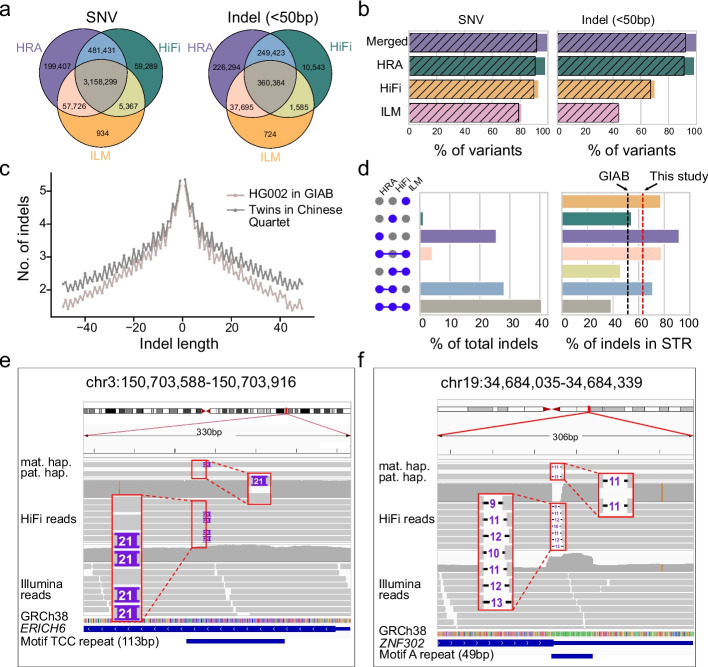
Small variant in the twins of the Chinese Quartet. **a** Overlap of SNVs and indels among ILM, HiFi, and HRA, respectively. **b** Bar plot depicts the percentage of ILM, HiFi, and HRA calls in SNV (left) and indel (right) benchmark, with gray stripes representing the percentages of calls supported by BGI reads. **c** indel length distribution of indels across HG002 in GIAB (v4.2.1) and twins in Chinese Quartet. **d** Left bar represents the percentages of indels in seven different combinations of three technologies. Right bars represent the percentages of indels at STR regions across different combinations of three technologies. The black and red dot lines refer to the percentages of indels at STR regions in whole benchmarking set of GIAB and this study. **e** IGV snapshot shows a heterozygous insertion at a TCC repeat. This insertion is detected by both HRA and HiFi reads. **f** IGV snapshot shows a homozygous deletion at a homopolymer region. This deletion is only reported by HRA

We obtained 3,962,453 SNVs and 886,648 indels for the monozygotic twins across chr1-chr22 and chrX (Fig. 2a), of which 91.9% (3,639,668) of SNVs and 92.1% (816,621) of indels were also called using BGI reads (Fig. 2b and Additional file 2: Fig. S13). Notably, HRA-based variant calling strategies accounted for 98.3% (3,896,863) of SNVs and 98.6% (873,796) of indels, while long-read HiFi mapping based approaches accounted for 93.5% (3,704,386) of SNVs and 70.1% (621,935) of indels. By contrast, Illumina short-read mapping based variant calling yielded 81.3% (3,222,326) of the total SNVs and 45.2% (400,388) of the indels.

As expected, the indel length distribution (Fig. 2c) of the twins is largely consistent with that of HG002 in GIAB (v4.2.1). Additionally, the sensitivities of the three technologies for indel detection increase accordingly as their sequence lengths increase (Additional file 2: Fig. S14). Notably, 25.5% (226,294) of indels could only be detected using HRAs, of which 91.8% were found in STR regions. In general, the HiFi and HRA methods detected more indels in complex regions than that of Illumina approach (Fig. 2d and Additional file 2: Figs. S15, S16). For example, a 21-bp heterozygous insertion of a TCC repeat in *ERICH6* was accurately identified by both HRAs and HiFi reads, but missed by Illumina data due to its shorter read length (Fig. 2e). Another example was a heterozygous deletion in a homopolymer region (a 49-bp A repeat) of *ZNF302* which was missed by both HiFi and Illumina reads but reported as homozygous deletion by HRAs (Fig. 2f and Additional file 2: Figs. S17, S18).

#### Large deletion and insertion benchmark construction

Structural variants affect more nucleotides and are generally more deleterious than SNVs and indels [3], although they are relatively rare compared to SNVs and indels. However, SV detection and benchmarking remain challenging. To overcome the biases of SV detection across different technologies, SVs from Illumina reads, HiFi reads, and haplotype-resolved assemblies were discovered, filtered, and merged. Illumina calls were generated by four callers, including Manta [62] (v1.6.0), Delly (v0.9.1), Lumpy (v0.2.13), and Pindel (v0.3). HiFi calls were produced by pbsv (v2.6.2), Sniffles [39] (v1.0.12), cuteSV [40] (v1.0.11), and SVision [23] (v1.3.6). Apart from read-alignment strategies, we also used five HRAs to discover SVs, with SVs supported by at least three assemblies included in the HRA callset (Additional file 2: Fig. S12).

**Fig. 3:**
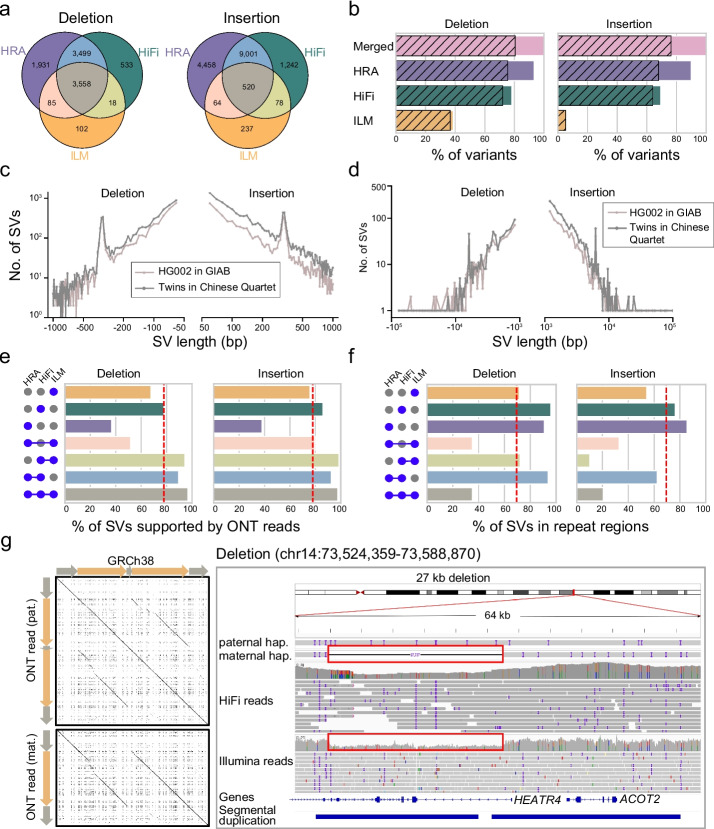
Simple structural variants in the twins of the Chinese Quartet. **a** Overlap of large deletions and insertions among ILM, HiFi, and HRA, respectively. **b** Bar plot depicts the percentage of ILM, HiFi, and HRA calls in the final simple SV benchmark, with gray stripes representing the supported percentages by ONT read. **c**, **d** Length distribution of tier 1 (v2.0) SVs across Chinese Quartet twins and HG002 (GIAB v0.6). **e** Bar plots show the percentages of variation supported by ONT reads in seven different combinations of three technologies. The red dotted lines represent the percentage of SVs that are supported by ONT reads across the entire benchmarking set. **f** Bar plots represent the percentages of SVs at STR regions in seven different combinations of three technologies. The red dotted lines represent the percentage of SVs that span repeat regions across the entire benchmarking set. **g** Dotplot and IGV snapshot shows a 27-kb deletion at a segmental duplication region

We obtained, in total, 9726 large (≥ 50 bp) deletions and 15,600 large (≥ 50 bp) insertions (≥ 50 bp) for the monozygotic twins across chr1-chr22 and chrX (Fig. 3a). HRAs accounted for 93.3% (9073) of deletions and 90.0% (14,043) of insertions, while HiFi reads accounted for 78.2% (7608) of deletions and 69.5% (10,841) of insertions, and Illumina calls 38.7% (3763) of deletions and 5.8% (899) of insertions. We found that 80.5% (7831) of deletions and 76.3% (11,908) of insertions could be independently supported by ONT reads (Fig. 3b). Similar to HG002 in GIAB (v0.6 in tier 1 regions), the SV length distribution of the twins displayed about 300 bp and 6 kb peaks related to SINE-Alu and LINE elements, respectively, suggesting the effective SV detection of our benchmark (Fig. 3c, d and Additional file 2: Fig. S19). When HiFi reads and HRAs were introduced to the analysis, we identified more SVs in repeat regions including VNTRs, simple repeats, and segmental duplications—and, overall, called a higher proportion of structural variants in the monozygotic twin daughters when compared to HG002 genome in GIAB (using the v0.6 tier 1 callset). We observed that SVs called using at least two of the three technologies (Illumina, HiFi, and HRAs) always achieved a higher validation ratio than those called using one technology independently (Fig. 3e, f and Additional file 2: Fig. S20, S21). In particular, there were 1931 deletions and 4485 insertions exclusively contributed by HRAs, and only 36.9% and 38.0% of those deletions and insertions, respectively, were supported by ONT reads. In addition, 91.0% and 85.7% of HRA-specific deletions and insertions, respectively, were located in repeat regions, where sequencing errors and multiple alignments of reads occur frequently. For example, HRAs identified a 27 kb maternal deletion within a segmental duplication of *HEATR4*, which was not reported in HiFi and Illumina read alignment-based callsets (Fig. 3g). To further validate this heterozygous deletion, we manually inspected all phased ONT reads. We found that 14 maternal and 2 paternal reads fully spanned this region, providing additional evidence for this event (Fig. 3g).

#### Complex Structural Variant (CSV) and inversion benchmark construction

**Fig. 4:**
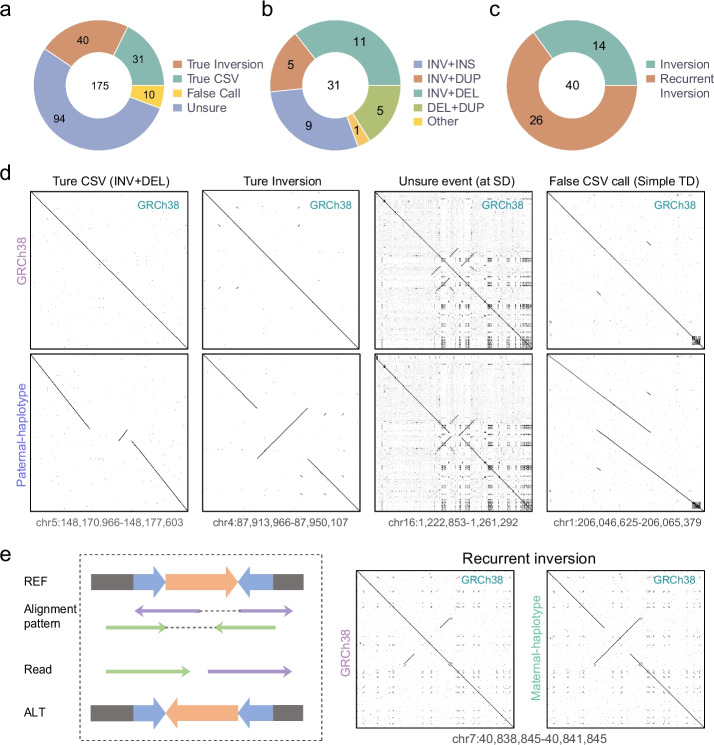
Complex structural variants and inversions in the twins of the Chinese Quartet. **a** Composition of complex SVs and inversions. **b**, **c** The pie plot shows the composition of different types of complex SVs (**b**) and inversions (**c**) in our benchmark. **d** Dotplots show examples of a true CSV, a true inversion, an unsure event, and a false CSV call. The true CSV example is a deletion-inversion. The unsure event example is reported in a segmental duplication region. The false CSV call example is a simple tandem duplication. **e** The diagram and dotplots shows the read alignment pattern in recurrent inversion

Detection of complex SVs and inversions was more complicated than that of simple variants due to ambiguous alignments, especially in repetitive regions. To build a benchmark for complex structural variants, we generated five callsets of complex SVs and inversions with HiFi reads and HRAs as input using Sniffles, SVision, cuteSV, pbsv, and PAV. Next, 175 nonredundant candidate variants from the merged callset were manually inspected and refined according to IGV snapshots and dotplots (Fig. 4a–c, Additional file 1: Table S9, Additional file 2: Figs. S22-S25, and Additional file 4). In the process of manual inspection, some simple variants, such as tandem duplication (Additional file 2: Fig. S24), were erroneously reported as CSVs caused by repeat content (Fig. 4d). We also found 53.7% of events that appeared in repeated regions, where we cannot confirm whether these were true CSVs or false calls due to the poor read mapping quality (Additional file 2: Fig. S25). After filtering these unsure events and false calls, we constructed a final set of 31 CSVs, of which 90.3% contained an inverted segment (Fig. 4b). We found that Sniffles, SVision, and cuteSV called 80.6–87.1% of CSVs, while PAV only called 32.3% (Additional file 2: Fig. S26). Only five CSVs were identified by all five callers, emphasizing the challenge of CSV detection.

We identified 40 inversions, of which 75% had allele frequencies larger than 0.5 in the HGSVC [33] callset (Additional file 1: Table S9). We observed that 26 (65%) of these inversions were flanked by inverted repetitive sequences and regarded these as recurrent inversions [63] (Fig. 4e and Additional file 2: Fig. S27). Notably, 92.3% (24) of recurrent inversions were discovered in more than 50% of HGSVC samples, while 50% (13) of recurrent inversions were present in all HGSVC samples. These findings suggest that majority of recurrent inversions are probably caused by mis-assembly of the reference genome in complex regions (Additional file 2: Fig. S28).

#### De novo and putative somatic mutation analysis

Compared to the trio design, the quartet samples are better suited to benchmarking de novo and somatic mutations that are sensitive to random errors in the experiment. We applied DeepVariant (v1.1.0) to the Illumina and HiFi reads of four samples. For each sequencing technology, the variant call files (gVCFs) from the four samples were merged and genotyped by glnexus (v1.2.7, https://github.com/dnanexus-rnd/GLnexus). Next, we removed variants present in either parent or with an allele frequency greater than 0.75 in either twin. Then, the variants shared by both twins were included in the candidate set for de novo mutations, while the variants specific to one twin daughter were included in the candidate set for putative somatic mutations. To further reduce the false positives, variants located in repeat regions, including STR, VNTR, SD, and SM, were excluded. Finally, we detected 68 de novo and 153 putative somatic mutations (Additional file 1: Table S10). Among the de novo mutations, 59 (53 SNVs and 6 indels) were confirmed through manual curation, while none of the putative somatic mutations were validated [64].

### Application of the variant benchmark

To ensure accurate identification of possible false negatives when using the benchmark, we defined the benchmark regions for the twins based on the haplotype-resolved assemblies (see the “Methods” section). The benchmark regions covered 92.43% of GRCh38 (approx. 2.80 Gbp in total, covering chr1-chr22 and chrX), which is comparable with HG002 in GIAB (v4.2.1, 2.75 Gbp).

**Fig. 5:**
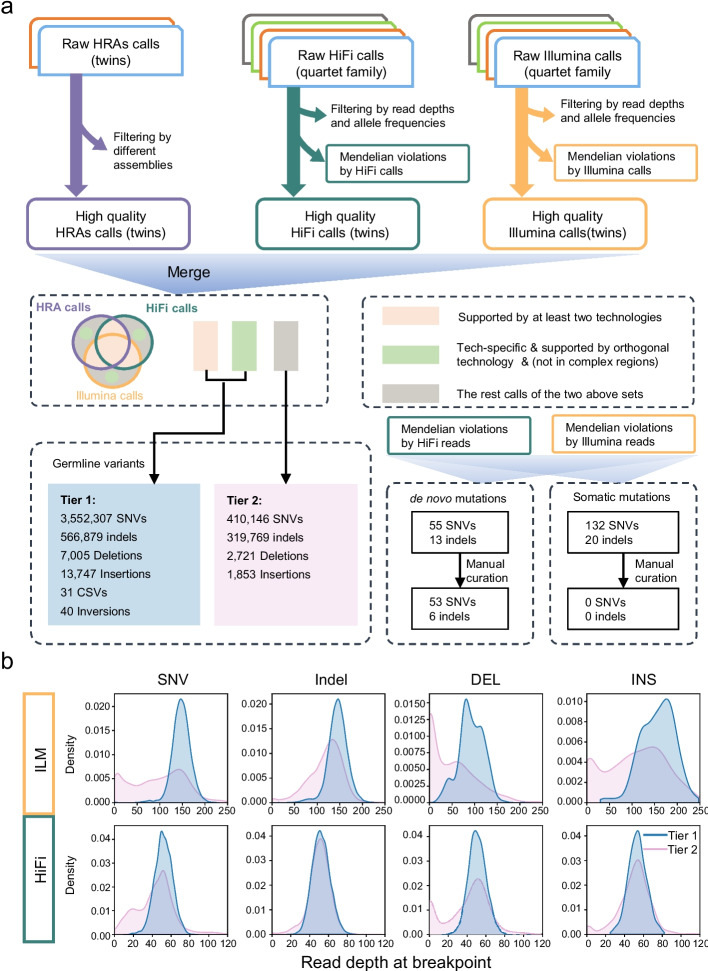
Summary and characteristics of the variant benchmarking set. **a** Summary of variant benchmarks in Chinese Quartet. **b** The density plots show the difference of variant characteristics between tier 1 and tier 2 calls in v2.0 of Chinese Quartet benchmarking set

In variant detection pipelines, complex regions like SD, SR, VNTR, and STR usually result in sequencing errors and multiple read alignments, particularly when using short read sequencing [65]. The long-read length and high base precision of HiFi and HRAs facilitated the detection of variants in complex regions that were not accessible for other technologies (Additional file 2: Figs. S29-S32). Accordingly, we stratified variants into tier 1 (high confidence) and tier 2 (relatively lower confidence) categories on basis of their supporting evidence (Fig. 5a). In v2.0 of Chinese Quartet, variants supported by at least two of the three technologies (Illumina, HiFi, and haplotype-resolved assemblies) were included in tier 1. For technology-specific variants, we included them in tier 1 if they were not located in repeat regions and supported by an orthogonal technology (BGI or ONT). Otherwise, they were included in tier 2. In v2.0 benchmark, tier 2 calls account for 10.4% of SNVs, 36.1% of indels, 28.0% of deletions, and 11.9% of insertions. As expected, in tier 2 callsets, 76.2% of SNVs, 97.8% of indels, 94.0% of deletions, and 84.1% of insertions were in repeat regions. Compared to tier 1 calls, we found that technology-specific calls always had abnormal read depths and low alignment rates as a consequence of repetitive regions (Fig. 5b and Additional file 2: Fig. S33). In v2.1 of the Chinese Quartet benchmark, similar to GIAB, we defined a more exclusive tier 1 to specifically address ultra-high-quality benchmarking in simple regions (Additional file 3).

We also found that variants in our benchmark were enriched (Wilcoxon rank-sum one-sided test, *P* < 2.8 × 10^−6^) in the proximal telomere of metacentric chromosomes instead of being randomly distributed about the genome (Additional file 2: Figs. S34, S35). Meanwhile, the densities of SNVs and indels were strongly correlated with the density of STR (SNV: *R* = 0.68, *P* = 6.53 × 10^−45^; indel: *R* = 0.83, *P* = 4.22 × 10^−82^), while the densities of large deletions and insertions were strongly correlated with the density of VNTR (deletion: *R* = 0.78, *P* = 5.14 × 10^−67^; insertion: *R* = 0.81, *P* = 1.21 × 10^−73^) (Additional file 2: Fig. S36). In our benchmark, we found that 27,665 SNVs, 1021 indels, 58 deletions, and 78 insertions affected coding DNA sequence (CDS) regions (Additional file 1: Table S11).

To facilitate the use of this benchmark, we provided a script to assess variants across various genome regions. Our script integrated *hap.py* (https://github.com/Illumina/hap.py) and truvari [66] and provided an option for users to analyze their methods in terms of different regions, such as segmental duplications and short tandem repeat regions. By comprehensively evaluating the performance of input variants in various genomic regions, developers can purposefully optimize their method, while users can choose the most appropriate caller for their needs. We also applied the initial callsets of the twins to the script and observed that all callsets achieved higher *F*-scores in tier 1 regions compared to tier 2 and other repetitive regions in v2.0 of the benchmark (Additional file 2: Fig. S37). In addition, manual inspection of the reported false positives and false negatives in tier 1 ensured that our benchmark follows the reliable identification of errors (RIDE) principle [5, 10, 64] (Additional file 1: Table S12 and Table S13).

### Assemblies and variant detection at different sequencing depths

**Fig. 6:**
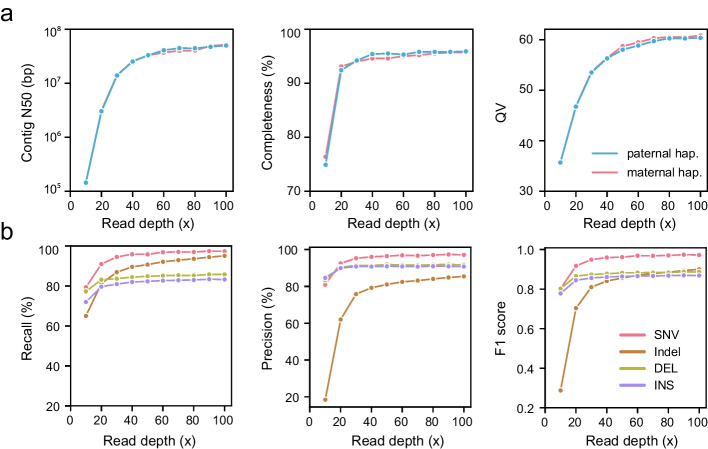
Quality of Chinese Quartet assemblies and accuracy of variant calling at different HiFi sequencing depths. **a** Contig N50 (left), completeness (middle), and QV (right) for paternal and maternal haplotypes across 10 × to 100 × HiFi sequencing depths. Completeness and QV are calculated by BUCSO and Merqury, respectively. **b** Recall, precision, and F1-score for SNVs, indels, large deletions, and insertions using assemblies with diverse HiFi sequencing depths (ranging from 10 × to 100 ×)

Sequencing depth was an important factor for both assembly and variant detection. To assess the effect of depth upon assembly and variant detection pipelines, samples with different sequencing depths (ranging from 10 × to 100 × coverage) were generated by downsampling the HiFi reads of the monozygotic twins. Initially, samples with different sequencing depths were assembled into haplotype-resolved assemblies by hifiasm [38]. The contig N50 of the two haplotypes flattened out with increasing sequencing depth and was maintained for more than 25 M at 40 × (Fig. 6a and Additional file 2: Table S14). The BUSCO completeness also increased rapidly and reached around 94% at 30 × (Fig. 6a). The accuracy of assemblies (QV) also increased steadily with increasing depth and remained stable from 60 × (Fig. 6a). To further evaluate the performance of variant detection with HRA in diverse sequencing depths, two haplotypes from different depths were used for variant detection with PAV [33]. Like the performance of assemblies, the recall, precision, and F1 score of variants also improved with increases in depth, reaching a plateau at 30 × (Fig. 6b and Additional file 1: Table S15). Taken together, these results suggest that 30 × HiFi reads could achieve optimal performance when used with appropriate assembly and variant detection pipelines.

### Decoding HLA regions with haplotype-resolved assemblies

**Fig. 7:**
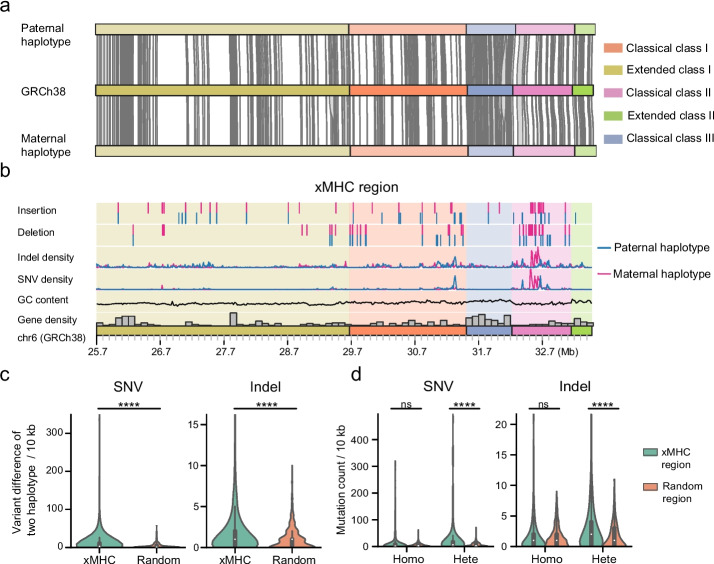
Assemblies and variants of the Chinese Quartet at the extended major histocompatibility complex region. **a** Alignment of paternal and maternal haplotypes to GRCh38 at the extended major histocompatibility complex (xMHC) region (chr6: 25,701,783–33,480,577). Both haplotypes covered the xMHC region with only one contigs. Gray links between haplotypes and GRCh38 are the protein coding genes resolved. **b** Genetic characteristics of two haplotypes. **c** Violin plot shows the variants difference between two haplotypes in 10 k bp windows. The variant difference in the xMHC region is significantly higher than that in other, randomly selected, regions (Wilcoxon rank-sum two-sided test; *P* < 0.0001). **d** Violin plot shows the heterozygous and homozygous variants count in 10 k bp windows. The number of heterozygous SNVs and indels in xMHC regions are significantly more than those in other random regions, while homozygous variants have no significant difference. *P*-value was calculated with Wilcoxon rank-sum two-sided test. ns, not significant; *P < 0.05; **P < 0.01; ***P < 0.001; ****P < 0.0001

Human leukocyte antigen (HLA) genes are important in cancer, autoimmune disease, infectious disease, and tissue transplantation [67]. To better understand the genetic features of human leukocyte antigen genes, we investigated the extended major histocompatibility complex [68] (xMHC) region of two twin daughters based on the haplotype-resolved assemblies and variant benchmarking set. We observed that both CQ-P and CQ-M covered the entire xMHC region in GRCh38 without any gap (Fig. 7a and Additional file 2: Fig. S38). In addition, 265 out of 271 protein-coding genes located within the xMHC regions were resolved by both CQ-P and CQ-M. We identified 13.5 kb and 19.0 kb of novel sequence compared to GRCh38 in paternal and maternal haplotypes, respectively. Our benchmarking set included 28,662 SNVs, 3725 indels, 61 large deletions, and 64 large insertions spanning 7.78 Mbp of the extended MHC region, in comparison to GIAB which used linked reads and long reads to call 22,368 small variants spanning 4.97 Mbp of classical MHC region [8]. Compared to classical class III regions, classical class I and II regions had a higher number of variant calls (Fig. 7b). We also discovered the difference of variants between two haplotypes in the xMHC region is higher than those in other regions (Fig. 7c). Furthermore, we discovered that the heterozygous SNVs and indels in the xMHC regions were significantly (*P* < 2.57 × 10^−10^) more prevalent than those in other regions, while homozygous variants showed no significant (*P* > 0.24) difference in prevalence (Fig. 7d), confirming the linkage disequilibrium of HLA regions [69].

## Discussion

Here, we report the generation of a Chinese Quartet, using these to construct a comprehensive catalogue of human genetic variants. The twin daughters of the Chinese Quartet could be regarded as two biological replicates, which facilitates additional cross validation and random error filtering compared to variant calling in a single sample or even in a trio. Moreover, the quartet family facilitate the detection of de novo and putative somatic mutations that are prone to false positives caused by random errors in both the trio and single sample designs.

Compared to the complete hydatidiform mole (CHM13), it is more challenging to decode the complete genome of a diploid sample. Nevertheless, 76% of the chromosome arms in our assemblies of monozygotic twins were gap-free from telomere to centromere (Additional file 1: Table S5). Meanwhile, seven and nine chromosomes of CQ-P and CQ-M were assembled at telomere-to-telomere levels, respectively (Additional file 1: Table S5). Although long-read technologies, including HiFi and ultra-long ONT reads, were applied in our assemblies, it was still difficult to distinguish two haplotypes of diploid samples in large repetitive regions, such as higher-order repeats in centromeres, original gaps in the assemblies, and novel sequencing regions (Additional file 4: Table S16). To obtain a high-quality assembly in these large repetitive regions, we randomly assigned the unmapped and unphased reads equally into two haplotypes in our assembly pipeline. Regions contributed to the assemblies by these uncertain reads are labeled (Additional file 4: Table S16) such that further validation with longer and more advanced reads may be performed in the future.

When including haplotype-resolved assemblies for benchmarking, more large-scale variants were detected due to the longer spanning length of HRAs on the genome [33] (Additional file 5: Table S17). Meanwhile, many variants in complex regions such as xMHC and segmental duplications were reported, which are difficult to detect using read-alignment based variant calling strategies. Another contribution of our benchmark is that, compared to previous studies [10, 21, 22, 70], we extend the set of variant types to include complex structural variants and de novo mutations. Nevertheless, our benchmark also has several limitations. Firstly, technology-specific variants should be subjected to further validation as it was difficult for current technologies to decode all complex regions unbiasedly, such as homopolymer regions and segmental duplications (Figs. 2f and 3g). Secondly, certain structural variants in our benchmark may be reported as multiple records at repetitive regions due to breakpoint shifts. Thirdly, we also observe that different methods of variant comparison or different representations of the same variant can lead to misjudgments of false positives and false negatives in benchmarking. Fourthly, since the samples used in this study are cell lines from healthy individuals, somatic and mosaic mutations have not been comprehensively analyzed in the benchmarking.

To deal with these limitations, the community should leverage advanced technologies to develop novel methods for variant benchmark validation, construction, and comparison. For instance, automated and unbiased methods for variant validation should is crucial to overcome the inefficiency and subjective factors introduced by manual curation. Furthermore, utilizing complete T2T assembly and human pangenome as reference genomes could effectively address some challenging regions that are ill-represented in GRCh37 and GRCh38. Pangenomes and graph-based variant detection, representation, and comparison methods should be applied in benchmarking processes, which will be highly beneficial in addressing challenges posed by highly repetitive regions such as the centromere. For the next phase of the Chinese Quartet Project, we will develop new algorithms and generate novel data to improve both the de novo assemblies and variant benchmarking set so as to facilitate resequencing projects of the Chinese Han population.

## Conclusions

The Chinese Quartet Project provides high-quality haplotype-resolved assemblies for the two monozygotic twins of the quartet family, each of which exceeds GRCh38 in contiguity and completeness, alongside a comprehensive variant catalogue for the twins. We expect these resources to aid in the ongoing improvement of sequencing technologies and variant calling pipelines, especially for complex variants, as well as giving insight into regions of the human genome technically challenging to assemble.

## Methods

### Sequencing data

The “Chinese Quartet” family, comprising a 60-year-old father (LCL7), 60-year-old mother (LCL8), and two 30-year-old monozygotic twin daughters (LCL5 and LCL6), was from the Fudan Taizhou cohort, which was approved as Certified Reference Materials (CRMs) by the State Administration for Market Regulation in China (GBW09900-GBW09903). The processes of cell line establishment, DNA extraction, and Illumina sequencing were described in prior studies [11, 13]. The four cell lines were also sequenced using BGI, PacBio, and ONT technologies (Additional file 3).

### Separation of reads by haplotype

To build haplotype-resolved assemblies for the monozygotic twins of the Chinese Quartet, we split HiFi and ONT reads into paternal (CQ-P) and maternal (CQ-M) haplotypes. Firstly, we obtained 3,249,650 single nucleotide variants (SNVs) and 404,882 indels of the family from a previous study [11]. The variants of the monozygotic twin daughters were phased using the “phase” command of whatshap [44] (v1.1) with parent–child information and each child’s HiFi reads. We then aligned the HiFi, ONT, and ultra-long ONT reads from each twin to GRCh38 with minimap2 (v2.20-r1061). We added haplotype tags to the aligned BAM files using the “haplotag” command of whatshap and assigned reads with haplotype tags to their respective haplotypes using the “split” command of whatshap. Reads which were either unassigned to a haplotype or unmapped to GRCh38 were randomly assigned to the two haplotypes.

### Genome assembly

As monozygotic twins are in general regarded as genetically identical with limited somatic mutations [43], we merged the phased reads from two twin samples for each haplotype to obtain high-quality haplotype-resolved genomes. For each haplotype of the monozygotic twin daughters, we assembled phased HiFi reads using hifiasm [38] (v0.15.5), hicanu [47] (v-r10117), and flye [46] (v2.8.3-b1695). Meanwhile, ONT regular and ONT ultra-long reads were assembled with flye [46] (v2.8.3-b1695) and shasta [45] (v0.7.0). Next, we identified the mis-assemblies and broke chimeric contigs with ragtag [48, 71] (v2.0.1). Then, we scaffolded the hifiasm contigs based on the human Telomere-to-Telomere genome [31] (CHM13-T2T v1.0) and closed the gaps of hifiasm scaffolds with other contigs [72, 73] (Additional file 3). Finally, the two haplotypes were polished with their corresponding HiFi reads using NextPolish [49] (v1.3.1).

### Evaluation of assembly accuracy, continuity and completeness

The two haplotype-resolved assemblies of the monozygotic twin daughters were evaluated on the basis of accuracy, continuity, and completeness. To assess the accuracy of the genome, we calculated the consensus quality value (QV) for each haplotype using Merqury [74] (v1.3), taking the Illumina reads of the family as input. Continuity was calculated on basis of contigs, contig N50, and the number of gaps. Three methods were applied to evaluate the completeness of CQ-P and CQ-M. First, we used BUSCO [57] (v5.1.3) with the “mammalia_odb10” database to calculate the proportion of complete BUSCO genes included in the assembly. Secondly, Merqury [74] (v1.3) was used to estimate the k-mer completeness of HRAs with Illumina sequencing data. Thirdly, we aligned both haplotypes to GRCh38 with minimap2, considering the coverage fraction to be a proxy of completeness.

### Genome annotation and identification of novel sequences

We used Liftoff [58] to annotate genes from the Gencode annotation (v38) of GRCh38 to both haplotypes. To annotate the novel genes, we aligned contigs of two haplotypes of the Chinese Quartet twins to GRCh38 with minimap2 [60] (v2.20-r1061) and winnowmap2 [75] (v2.03). Thereafter, the sequences labeled by hard-clip (H), soft-clip (S), and insertion (I) in BAM files were extracted and aligned to GRCh38 again to remove the duplicate or transitional sequences of the genome. Sequences unmapped after this second alignment step were considered novel sequences. These novel sequences were repeat masked using RepeatMasker (v4.1.2-p1, http://www.repeatmasker.org) and annotated by Augustus [59] (v3.4.0).

### Variant detection using Illumina reads

We downloaded 3,249,650 SNVs and 404,882 indels (< 50 bp) previously called in Chinese Quartet using the Illumina [11]. To complement this dataset with structural variants, we aligned these Illumina reads to GRCh38 using bwa [76] (v0.7.17), marking duplicated reads with biobambam2 [77] (v2.0.182). Variants were called using Manta [62] (v1.6.0), Delly [78] (v0.9.1), Lumpy [79] (v0.2.13), and Pindel [80] (v0.3) with parameters as described in the Additional file 3. We retained only those SVs at least 50 bp long and supported by at least 30 reads. We retained SVs following the Mendelian rules (Additional file 3). High-quality variants from four callers were then compared and merged by Jasmine [81] (v1.1.5) for each SV type (deletion and insertion), respectively. Finally, variants supported by at least two of the four callers were retained for the final benchmarking set.

### Variant detection of using HiFi reads

We aligned HiFi reads to GRCh38 using minimap2 [60] (v2.20-r1061) and then detected small variants for each sample using DeepVariant [61] (v1.1.0). The gVCFs of four samples were merged and genotyped by glnexus (v1.2.7, https://github.com/dnanexus-rnd/GLnexus). SNVs and indels were phased using whatshap [44] (v1.1), taking the parent–child information and children’s HiFi reads as input. To obtain high-quality SNVs and indels, we filtered variants to (i) remove those with allele frequencies < 0.2, read depth < 25, read depth > 75 or length > 49 bp; (ii) remove those violating the Mendelian rule, (Additional file 3), and (iii) retain only those where both twins had identical genotypes.

To obtain high-quality SV calls, we utilized four popular callers, pbsv (v2.6.2), Sniffles [39] (v1.0.12), cuteSV [40] (v1.0.11), and SVision [23] (v1.3.6), to independently identify SV events. Similar to Illumina reads, we retained only those SVs at least 50 bp long and supported by at least 15 reads. We retained only those SVs following the Mendelian rule and supported by at least two of four callers for the final benchmarking set.

### Variant detection using haplotype-resolved assemblies

We aligned echo of the five HRAs to GRCh38 using minimap2 [60] (v2.20-r1061) and called variants using the PAV [33] (v1.1.0) pipeline with default parameters. SNVs and indels were called using the three HiFi assemblies, with only variants identically called in all three retained for the final benchmarking set. SVs were called using both the HiFi and ONT assemblies. We retained for the final benchmarking set only those variants which were identically called in at least two assemblies.

### Curation of complex structural variants

In the initial SV callsets generated by HiFi and haplotype-resolved assemblies, SVs label as either “INV,” “complex SV,” or multiple SV types were extracted as candidate CSVs. To manually curate these candidates, the sequencing alignments of Illumina reads, HiFi reads, and HRAs in candidate regions were first visualized by IGV [82]. Dotplots between the HRAs and the reference genome in candidate regions were also generated by Gepard [83]. For a given candidate locus, we then manually inspected its IGV snapshots and the associated dotplots to determine the presence of the variant and its type [64] (Additional file 1: Table S9).

### Detection of de novo and putative somatic mutations

We utilized the Illumina and HiFi reads of four samples to detect de novo and somatic mutations of the monozygotic twin daughters relative to their parents. To obtain de novo and putative somatic calls, the variants that violated the Mendelian rule were extracted and the variants shared by both twins included in the candidate set for de novo mutation. Variants specific to one twin daughter were included in the candidate set for putative somatic mutation. To further reduce the false positives, variants located in repeat regions, including STR, VNTR, SD, and SM, were excluded. All candidate mutations were manually inspected according to the IGV snapshots of the Illumina and HiFi reads (Additional file 1: Table S10).

### Definition of benchmark regions

To define the benchmark regions, we first mapped the two haplotypes from different assemblers to GRCh38, retaining only those regions covered by both haplotypes. The gap regions of GRCh38, low confidence regions (LowConfidenceFilter.bed.gz, downloaded from HGSVC ftp), and regions with abnormal read depth (exclude.cnvnator_100bp.GRCh38.20170403.bed, downloaded from HGSVC ftp) were removed.

### Construction of variant call benchmarking set

SNVs and indels (< 50 bp) called using Illumina, HiFi, and HRAs were normalized and merged with bcftools (v1.13) and large deletions and insertions (≥ 50 bp) were compared and merged using Jasmine [81] (v1.1.5). Variants at centromeres, low confidence regions, copy number abnormal regions, and Y chromosomes were excluded in the final benchmark. Centromere regions were obtained from UCSC Table Browser (https://genome.ucsc.edu/cgi-bin/hgTables). The BED file of low confidence regions was downloaded from http://ftp.1000genomes.ebi.ac.uk/vol1/ftp/data_collections/HGSVC2/technical/filter/20210127_LowConfidenceFilter/LowConfidenceFilter.bed.gz. The BED file containing copy number abnormal regions was downloaded from http://ftp.1000genomes.ebi.ac.uk/vol1/ftp/data_collections/HGSVC2/technical/tech-support-files/exclude.cnvnator_100bp.GRCh38.20170403.bed.

To evaluate the quality of SNVs and indels in our benchmark, BGI reads were aligned to GRCh38 with bwa (v0.7.17-r1188), and DeepVariant [61] used to call SNVs and indels. ONT reads were aligned to the reference genome and four callers—pbsv (v2.6.2), Sniffles [39] (v1.0.12), cuteSV [40] (v1.0.11), and SVision [23] (v1.3.6)—were used to call variants. We retained only those SVs supported by at least 15 reads and 2 callers. In the v2.0 benchmarking set, variants that were supported by at least two of Illumina, HiFi, and haplotype-resolved assemblies were included in the tier 1 callset. For technology-specific variants, we included them in tier 1 if they were not located in repeat regions (STR, VNTR, SM, and SD) and supported by either BGI or ONT reads. Otherwise, they were included in tier 2 callset (Fig. 5a). In addition, to facilitate accurate benchmarking in simple regions, we have also defined a more conservative tier 1 in v2.1 of the benchmark (Additional file 3).

### Variant annotation

Repeat regions of GRCh38, including segmental duplications (SDs), simple repeats (SRs), and variable number tandem repeats (VNTRs), were downloaded from the UCSC table browser (https://genome.ucsc.edu/cgi-bin/hgTables). Short tandem repeats (STRs) of GRCh38 were generated using the “scan” command in msisensor-pro [84], taking the GRCh38 genome as input. A variant was annotated to a repeat region if it overlapped (by at least 1 bp) with a repeat region. Variants were also annotated by the Ensembl Variant Effect Predictor [85] (v104.3).

### Utility of Chinese Quartet benchmarking set

To facilitate the utility of this benchmark, we provided a snakemake workflow (https://github.com/xjtu-omics/ChineseQuartetGenome/tree/main/benchmarks) for evaluating variants across various genomic regions. The workflow combines *hap.py* (https://github.com/Illumina/hap.py) and truvari [66] to provide an evaluation report for each input VCF, quantifying precision, recall and *F*-score relative to the Chinese Quartet variants. Users can customize the workflow by amending the config file to implement their own programs as well as evaluating their input VCFs not only across the entire benchmarking region but in custom regions. For instance, users can evaluate a tool tailored to variant calling in challenging regions or particular variant types.

### Evaluating assembly and variant calling performance at different sequencing depths

To assess the quality of genome assembly step and subsequent variant calling at different sequencing depths, we randomly downsampled the HiFi reads of both monozygotic twins to depths of 10 to 100-fold coverage with increments of 10-fold. Seqtk (v1.3, https://github.com/lh3/seqtk) was used to downsample the sequences. We then assembled the simulated samples at each sequencing depth with hifiasm [38] (v0.15.5). Each assembly was evaluated for accuracy, completeness, and continuity, as detailed above. The variants at each sequencing depth were called using PAV [33] (v1.1.0) with two aligners, LRA [86] and minimap2. To evaluate variant calls, only those calls supported by both aligners in the PAV pipeline were retained for analysis. We considered variants supported by both the benchmark and simulated sample as “true positive” (TP) calls. Variants only supported by the simulated sample or benchmark were labeled as “false positive” (FP) and “false negative” (FN) calls, respectively. Finally, variant calling was evaluated on the basis of recall, precision, and F1 scores as given by Eqs. 1, 2, 3, respectively.

1 $$Recall=\frac{TP}{TP+FP}$$

2 $$Precision=\frac{TP}{TP+FN}$$

3 $$F1\ score=2*\frac{Recall\ \times\ Precision}{Recall\ +\ Precision}$$

### Supplementary Information

**Additional file 1: Table S1.** Sequencing summary of the Chinese Quartet. **Table S2.** Summary of phased reads from two haplotypes. **Table S3.** Summary statistics of the Chinese Quartet assemblies. **Table S4.** Assembly summary of the Chinese Quartet and other samples. **Table S5.** Distribution of contigs in Chinese Quartet assemblies. **Table S6.** The aligned fraction to GRCh38 of the Chinese Quartet haplotypes. **Table S7.** Completeness summary of our assemblies and other public papers. **Table S8.** Annotations of novel genes in Chinese Quartet. **Table S9.** Summary of Complex SVs and inversion of Chinese Quartet. **Table S10.** Information of de novo and putative somatic mutation. **Table S11.** Summary of VEP annotations for benchmarks. **Table S12.** Manual inspection result of 20 random false positives and false negatives for each variant type. **Table S13.** Detailed results of manual curations in Table S12. **Table S14.** Assembly performance at different sequencing depths of the Chinese Quartet. **Table S15.** Variant detection performance at different sequencing depths of the Chinese Quartet.

**Additional file 2: Fig. S1.** Length distribution of ONT reads in this study. **Fig. S2.** Haplotype-resolved assembly pipeline for the Chinese Quartet twins. **Fig. S3.** Alignments of paternal (top) and maternal (bottom) haplotypes to GRCh38. **Fig. S4.** Comparison between haplotype-resolved assemblies of the Chinese Quartet twin daughters and GRCh38 as well as CHM13. **Fig. S5.** Overview of the gaps in the assemblies of Chinese Quartet twin daughters. **Fig. S6.** Bar plots show the ratio of the number of abnormal bins to total the number of bins at both (a) chr17:21,523,754–22,371,820 (the regions covered in Fig. 1b) and (b) chr8: 6,281,267–13,968,020 (the region covered in Fig. 1c). **Fig. S7.** Dotplots between the Chinese Quartet twins assemblies and both the GRCh38 and CHM13-T2T (v2.0) at a region near the centromere of chromosome 17 (chr17:21,523,754-22,371,820). **Fig. S8.** Dotplots between the twins of the Chinese Quartet assemblies and both GRCh38 and T2T genomes at chromosome 8p23.1. **Fig. S9.** Circos plots show the characteristics of the paternal (A) and maternal (B) haplotypes of the Chinese Quartet twin daughters. **Fig. S10.** Distribution of novel sequence distribution in CQ-P (left) and CQ-M (right). **Fig. S11.** SNV and indel detection and validation pipelines. **Fig. S12.** Structural variant detection and validation pipelines. **Fig. S13.** Validated percentage of SNVs and indels across seven different combinations of three technologies. **Fig. S14.** Indel length distributions of HG002 and three technologies calls of Chinese Quartet twin daughters. **Fig. S15.** SNV rate in repeat regions across different combinations of three technologies. **Fig. S16.** Indel rate in repeat regions across different combinations of three technologies. **Fig. S17.** IGV show the alignment of HRAs, HiFi reads, and Illumina reads to the reference genome (GRCh38) in a 49bp homopolymer region. **Fig. S18.** Distribution analysis in homopolymer regions. **Fig. S19.** SV length distributions of HG002 and three callsets of Chinese Quartet twin daughters. **Fig. S20.** Large deletion rate in repeat regions across different combinations of three technologies. **Fig. S21.** Large insertion rate in repeat regions across different combinations of three technologies. **Fig. S22.** True CSV (DEL+INV) example at chr5:148,170,966-148,177,603 a-c, Dotplots show the comparisons of sequence between GRCh38 and GRCh38, GRCh38 and paternal haplotype, as well as GRCh38 and maternal haplotype at this locus. d, IGV snapshot shows the alignments of haplotype-resolved assemblies, HiFi reads, and Illumina reads at this locus. **Fig. S23.** True inversion example at chr4:87,913,966-87,950,107. **Fig. S24.** Tandem duplication were reported as CSV at chr1:206,046,625-206,065,379. **Fig. S25.** Unsure CSV in repeat regions at chr16:1,222,853, 1,261,292. **Fig. S26.** Complex SVs and inversions in benchmarks. **Fig. S27.** Recurrent inversion at chr7:40,838,845-40,841,845. **Fig. S28.** Violin plot shows the frequency of inversion in the HGSVC dataset. **Fig. S29.** F-scores of initial variants compared to the v2.0 benchmark of Chinese Quartet twin daughters. ILM_GB means the Illumina callset of the Chinese Quartet twin we obtain from the published paper (10.1186/s13059-021-02569-8). **Fig. S30.** Bar plots show the percentage of technology specific calls in v2.0 benchmark. **Fig. S31.** Variant features in different types of regions. **Fig. S32.** Benchmark evaluation of Chinese Quartet twin daughters. **Fig. S33.** The density plots show the difference in variant characteristics between high-confidence and technology-specific calls. **Fig. S34.** Variant distribution of Chinese Quartet at the telomere. **Fig. S35.** Variant distribution of Chinese Quartet at telomere and centromere. **Fig. S36.** STR/VNTR distribution and variant breakpoint correlation. **Fig. S37.** Diagram for benchmark utility. **Fig. S38.** IGV snapshot shows Chinese Quartet twins’ assemblies to GRCH38 in xMHC regions.

**Additional file 3: Supplementary notes.** In this additional file, we provide detailed processes of data generation, genome assembly, and construction of variant benchmarks [100, 101].

**Additional file 4: Table S16.** Known potentially problematic regions in our genome.

**Additional file 5: Table S17.** Summary of repeats in the benchmark regions.

## Data Availability

All raw data are available on the Quartet Data Portal ((http://chinese-quartet.org/) under the Administrative Regulations of the People’s Republic of China on Human Genetic Resources. All raw sequencing reads, assemblies, and variant benchmarks have also been deposited in the Genome Sequence Archive [87] at the National Genomics Data Center, Beijing Institute of Genomics, Chinese Academy of Sciences/China National Center for Bioinformation with BioProject IDs of PRJCA012291 [88] and PRJCA012423 [89]. The pipelines for genome assembly, merging, evaluation, and variant detection are available on GitHub [72, 90–93] and Zenodo [73, 94–97] under the MIT licenses. Additional data and codes supporting this article are disclosed on Github page [98] and Zenodo [64, 99], also under the MIT licenses.
