# Supplementary material for: Haplotype-resolved assemblies and variant benchmark of a Chinese Quartet
Source: Genome Biol. 2023 Dec 4;24:277. doi: 10.1186/s13059-023-03116-3 (PMC10694985; doi:10.1186/s13059-023-03116-3)
Supplement: Supplementary file 2 — Additional file 2: Fig. S1. Length distribution of ONT reads in this study. Fig. S2. Haplotype-resolved assembly pipeline for the Chinese Quartet twins. Fig. S3. Alignments of paternal (top) and maternal (bottom) haplotypes to GRCh38. Fig. S4. Comparison between haplotype-resolved assemblies of the Chinese Quartet twin daughters and GRCh38 as well as CHM13. Fig. S5. Overview of the gaps in the assemblies of Chinese Quartet twin daughters. Fig. S6. Bar plots show the ratio of the number of abnormal bins to total the number of bins at both (a) chr17:21,523,754–22,371,820 (the regions covered in Fig. 1b) and (b) chr8: 6,281,267–13,968,020 (the region covered in Fig. 1c). Fig. S7. Dotplots between the Chinese Quartet twins assemblies and both the GRCh38 and CHM13-T2T (v2.0) at a region near the centromere of chromosome 17 (chr17:21,523,754-22,371,820). Fig. S8. Dotplots between the twins of the Chinese Quartet assemblies and both GRCh38 and T2T genomes at chromosome 8p23.1. Fig. S9. Circos plots show the characteristics of the paternal (A) and maternal (B) haplotypes of the Chinese Quartet twin daughters. Fig. S10. Distribution of novel sequence distribution in CQ-P (left) and CQ-M (right). Fig. S11. SNV and indel detection and validation pipelines. Fig. S12. Structural variant detection and validation pipelines. Fig. S13. Validated percentage of SNVs and indels across seven different combinations of three technologies. Fig. S14. Indel length distributions of HG002 and three technologies calls of Chinese Quartet twin daughters. Fig. S15. SNV rate in repeat regions across different combinations of three technologies. Fig. S16. Indel rate in repeat regions across different combinations of three technologies. Fig. S17. IGV show the alignment of HRAs, HiFi reads, and Illumina reads to the reference genome (GRCh38) in a 49bp homopolymer region. Fig. S18. Distribution analysis in homopolymer regions. Fig. S19. SV length distributions of HG002 and three callsets of Chinese Qua [file 13059_2023_3116_MOESM2_ESM.pdf]

Supplementary figures for

Haplotype-resolved assemblies and variant benchmark of a Chinese Quartet

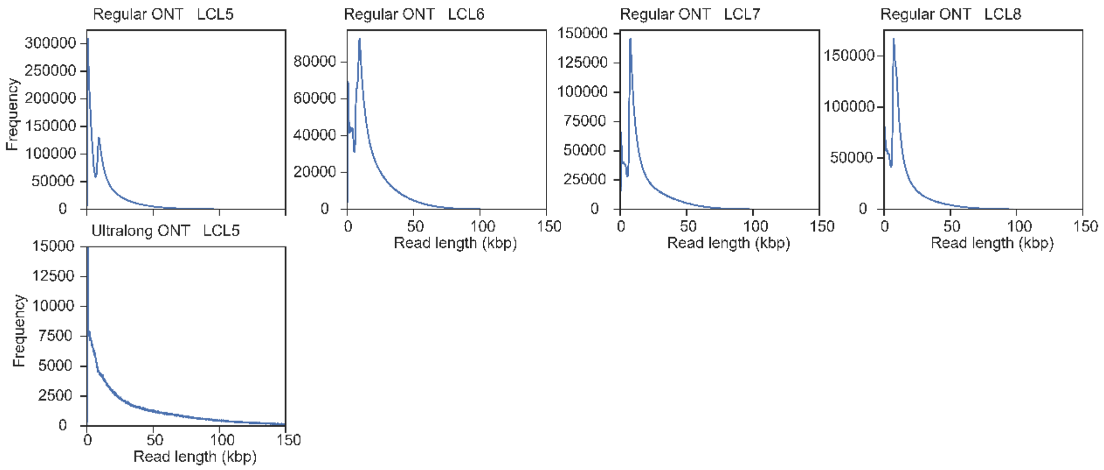

Fig. S1 Length distribution of ONT reads in this study.

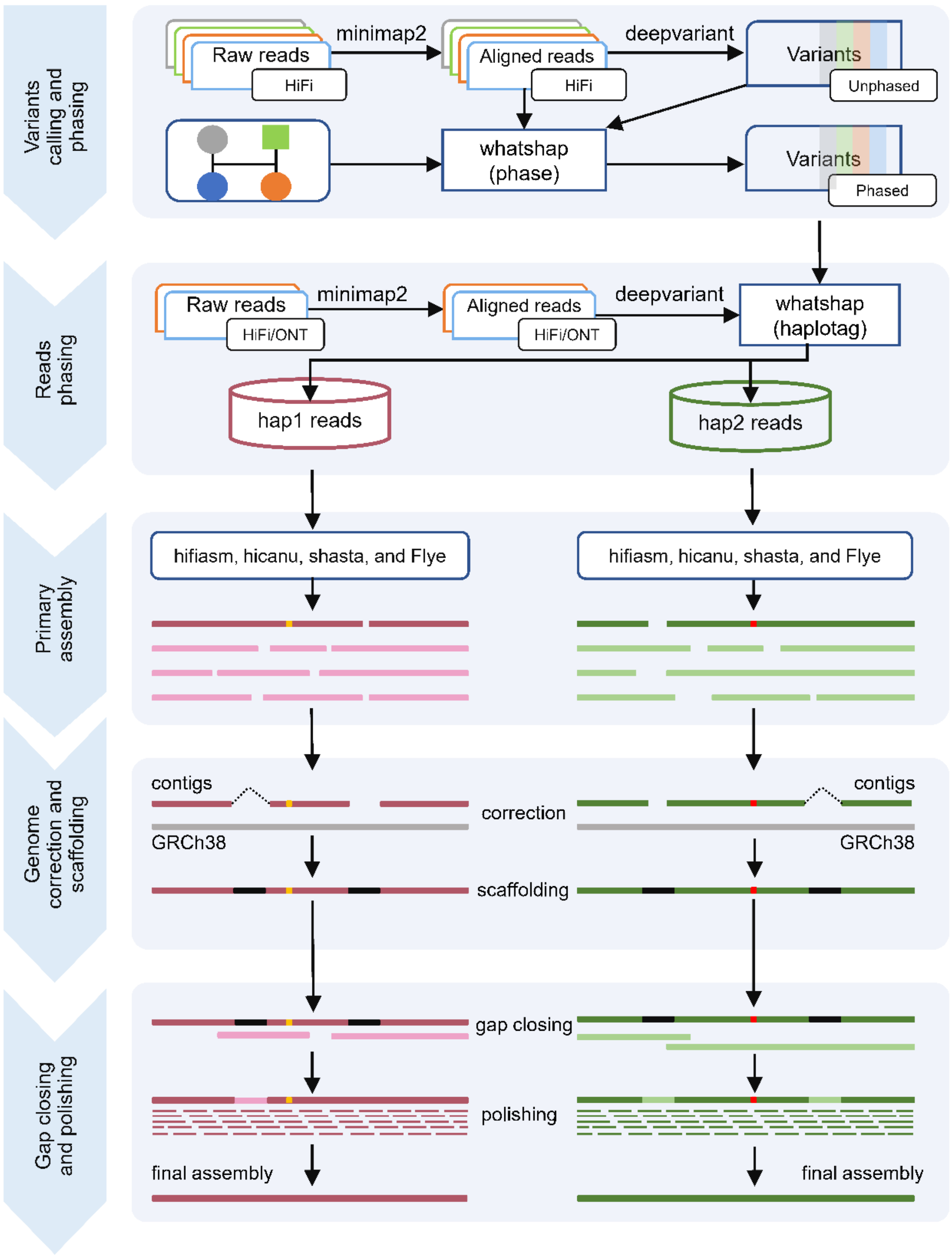

Fig. S2 Haplotype-resolved assembly pipeline for the Chinese Quartet twins.

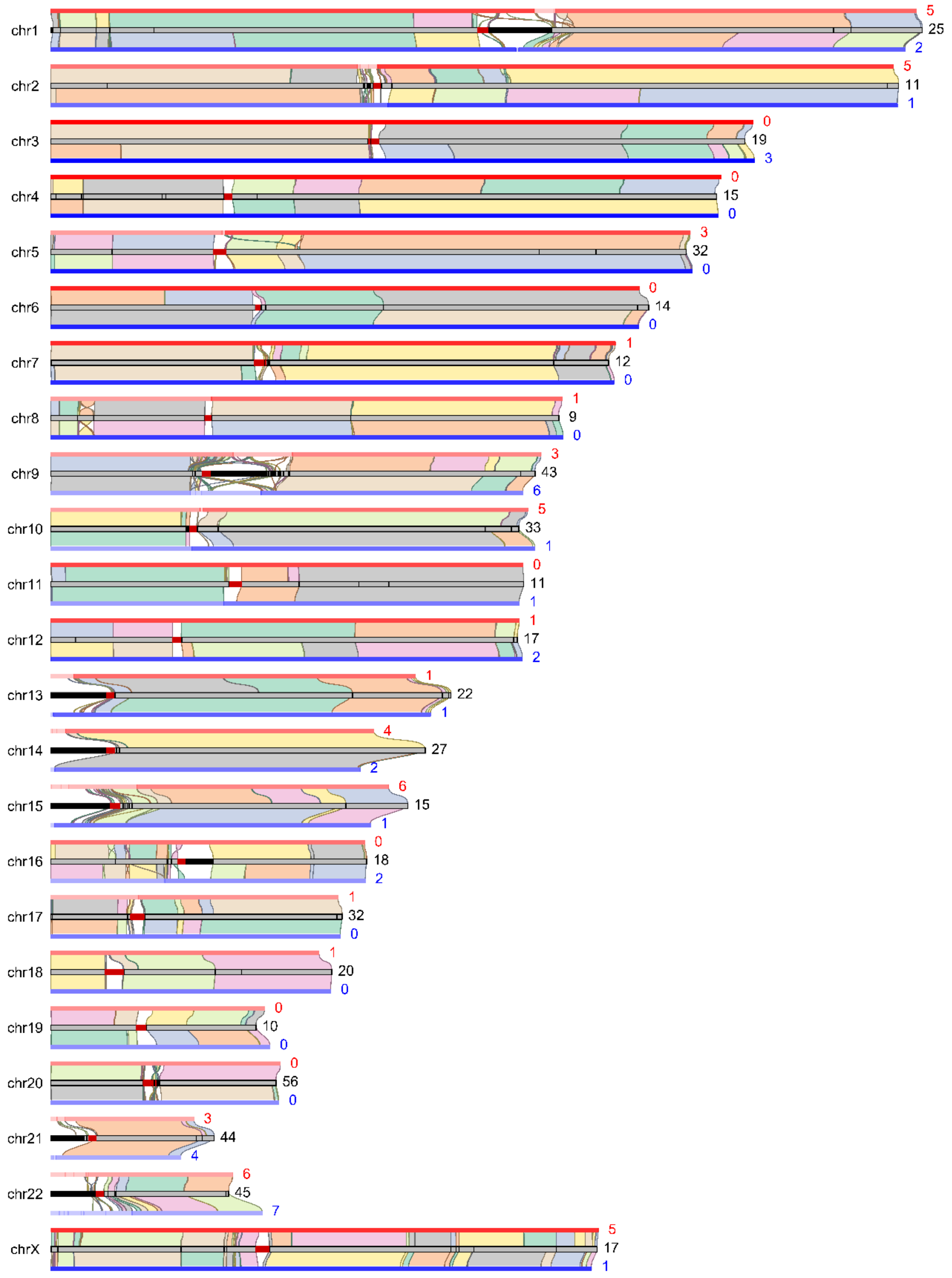

Fig. S3 Alignments of paternal (top) and maternal (bottom) haplotypes to GRCh38. The color shading of two haplotypes represents the contig size, with darker segments for longer contigs. The numbers of the gaps in CQ-P, CQ-M, and GRCh38 are labelled on the right of the chromosomes.

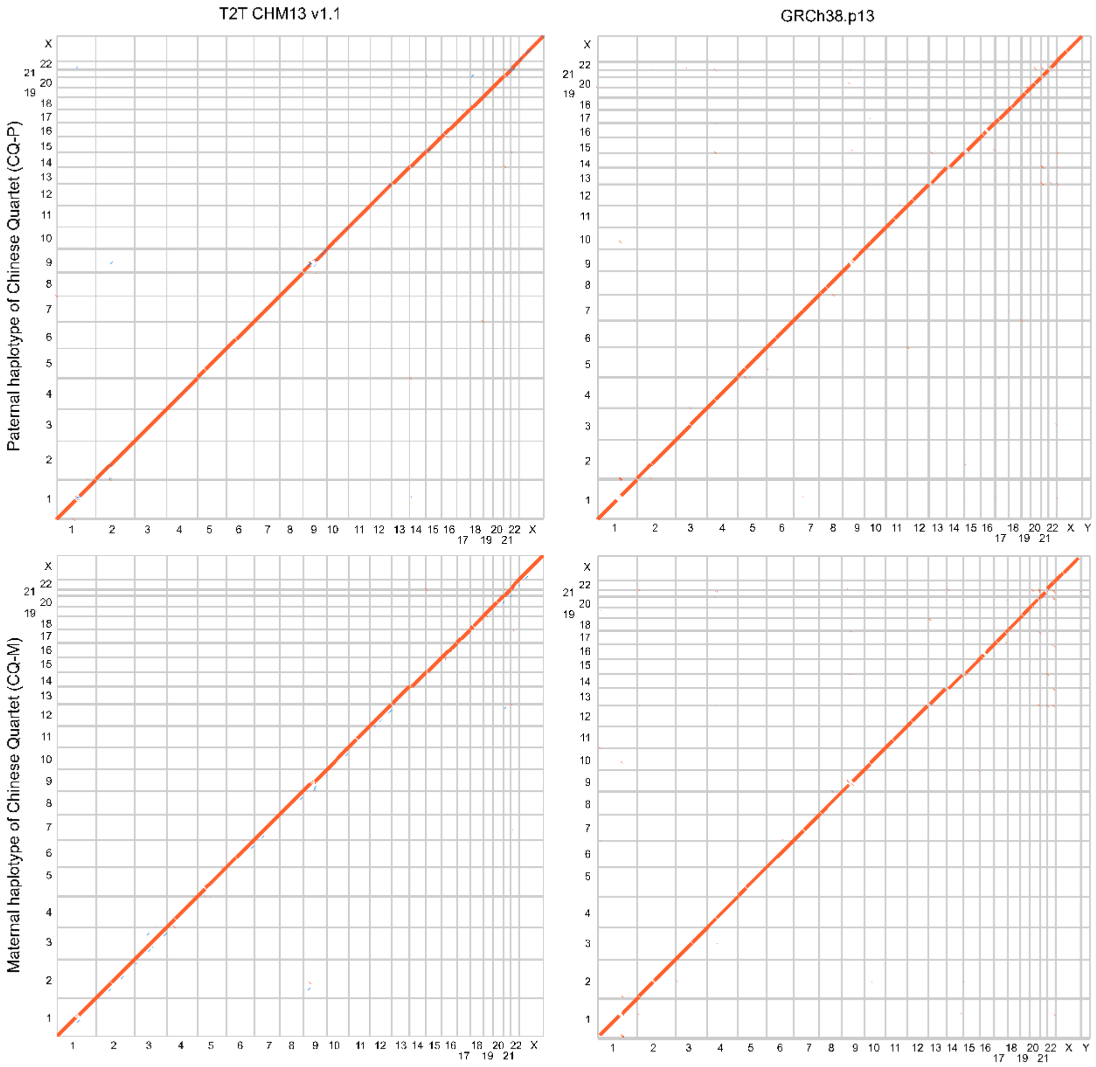

Fig. S4 Comparison between haplotype-resolved assemblies of the Chinese Quartet twin daughters and GRCh38 as well as CHM13. Dotplots represent the co-linearity between CQ-P/CQ-M and GRCh38/CHM13.

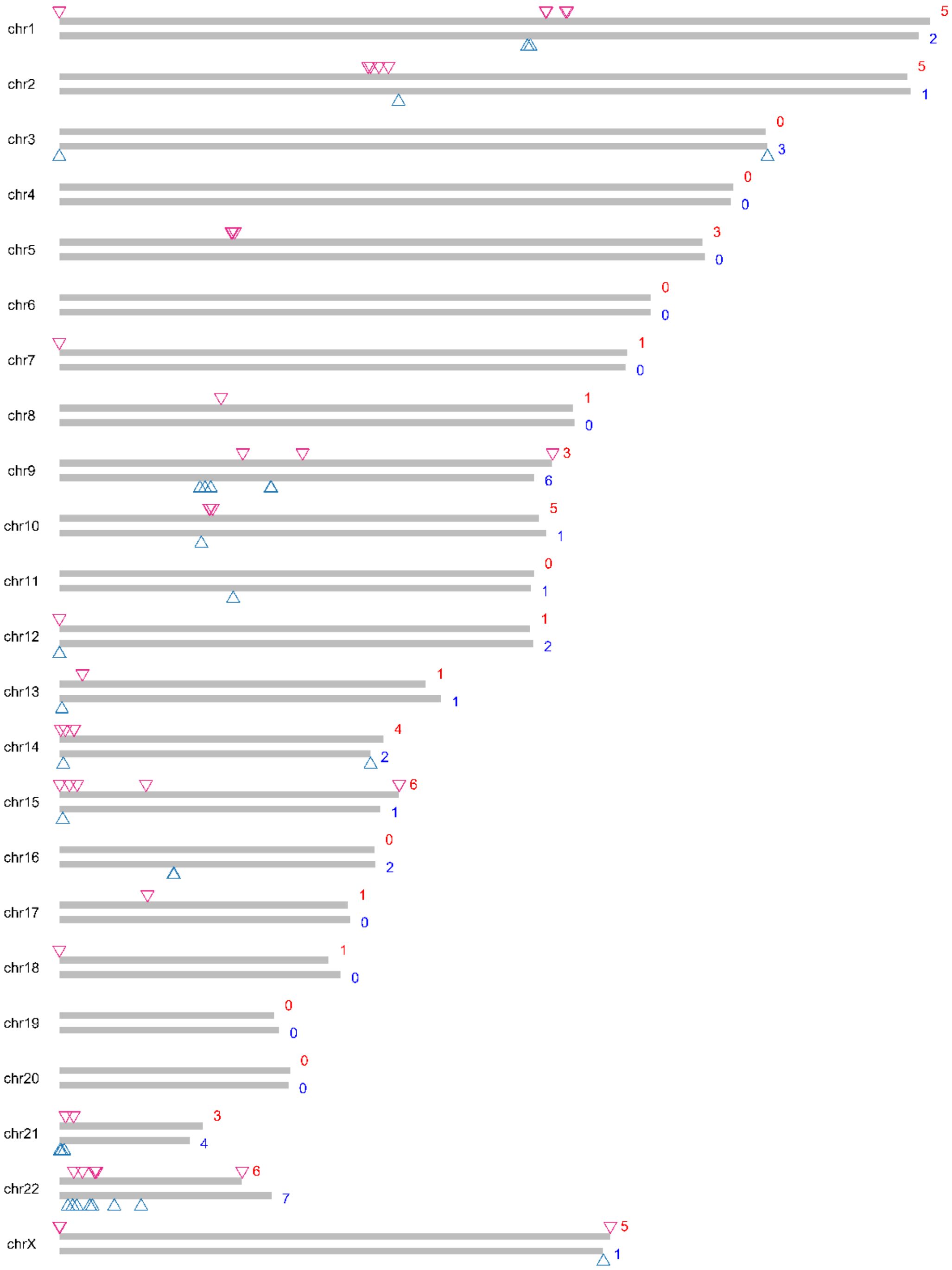

Fig. S5 Overview of the gaps in the assemblies of Chinese Quartet twin daughters. Triangles denote the locations of gaps on CQ-P (top) and CQ-M (bottom). The gap numbers of CQ-P and CQ-M are labelled on the right of the ideogram.

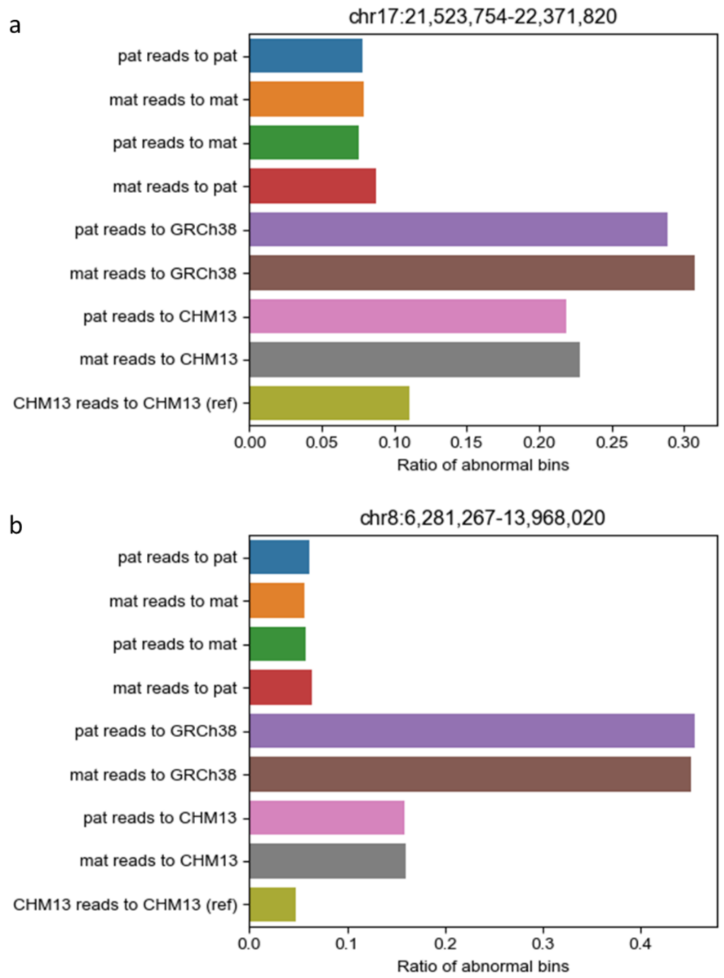

Fig. S6 Bar plots show the ratio of the number of abnormal bins to total the number of bins at both (**a**) chr17:21,523,754–22,371,820 (the regions covered in Fig. 1b) and (**b**) chr8: 6,281,267–13,968,020 (the region covered in Fig. 1c). T-axis labels take the form "A reads to B" and refer to the abnormal read depths when aligned reads of haplotype A to genome B. For example, "pat reads to CHM13" means aligned reads from the paternal haplotype (Chinese Quartet twins) to the CHM13 genome.

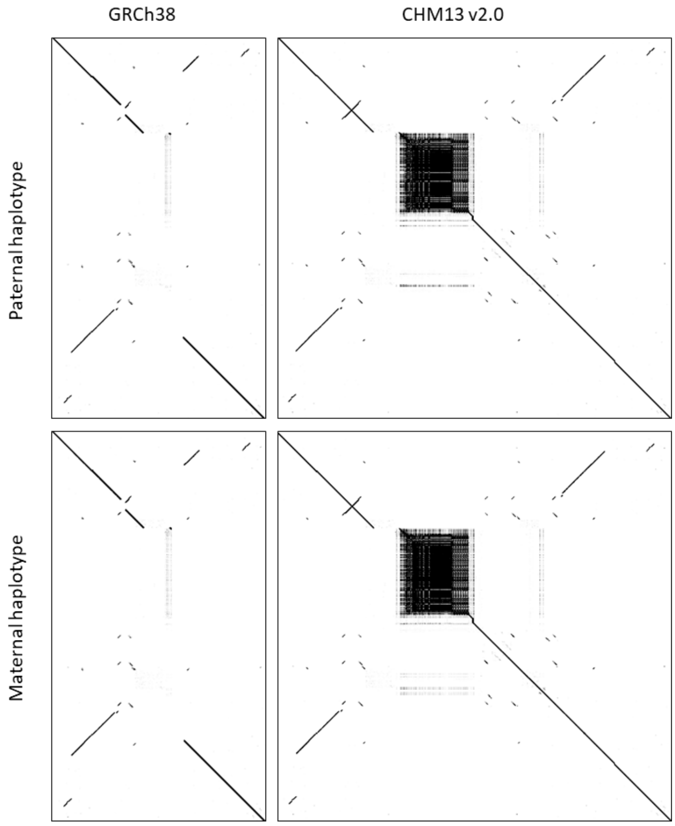

Fig. S7 Dotplots between the Chinese Quartet twins assemblies and both the GRCh38 and CHM13-T2T (v2.0) at a region near the centromere of chromosome 17 (chr17:21,523,754-22,371,820).

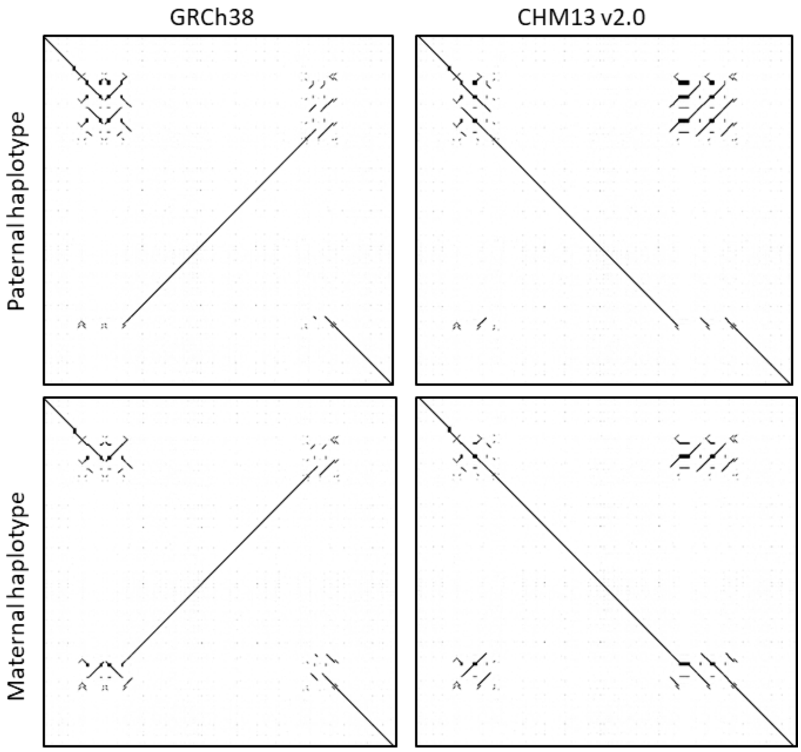

Fig. S8 Dotplots between the twins of the Chinese Quartet assemblies and both GRCh38 and T2T genomes at chromosome 8p23.1. Dotplots demonstrate that both paternal and maternal haplotypes have a ~ 4M inversion at chromosome 8 when compared to GRCh38 but are consistent with the sequences from the T2T genome.

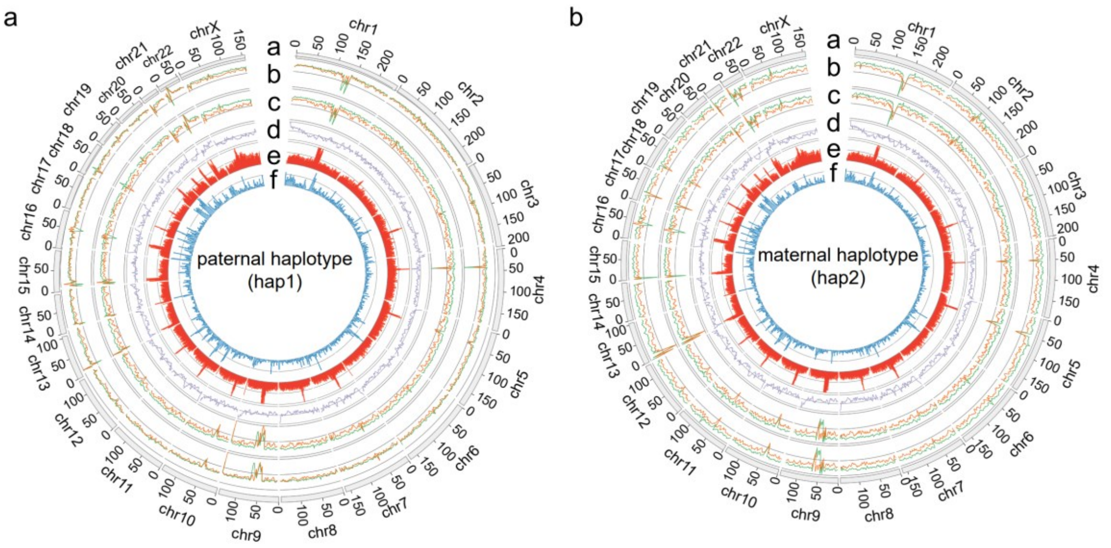

Fig. S9 Circos plots show the characteristics of the paternal (A) and maternal (B) haplotypes of the Chinese Quartet twin daughters. Track a denotes the ideogram. Track b and c represent the read depths of LCL5 (b) and LCL6 (c), respectively (orange for HiFi and green for ONT). Tracks d to f show the GC content, repeat density, and gene density, respectively.

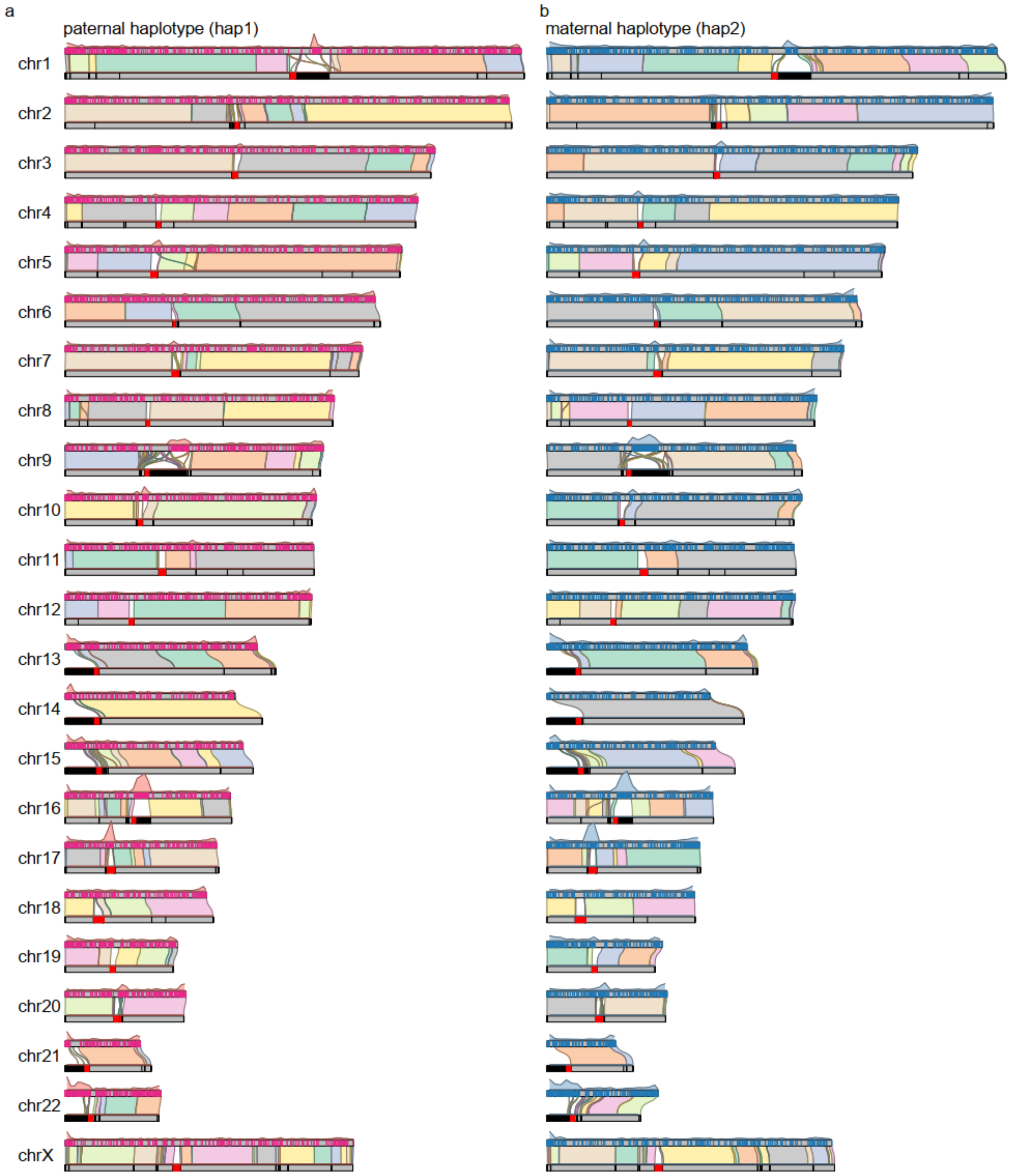

Fig. S10 Distribution of novel sequence distribution in CQ-P (left) and CQ-M (right). Alignments between haplotype (top) and GRCh38 (bottom) are represented by the links. The novel sequences of the haplotype are labeled by rectangles on the ideogram. Density plot shows the distribution of the novel sequence. Novel sequences are enriched in the centromeric and acrocentric regions of the genome.

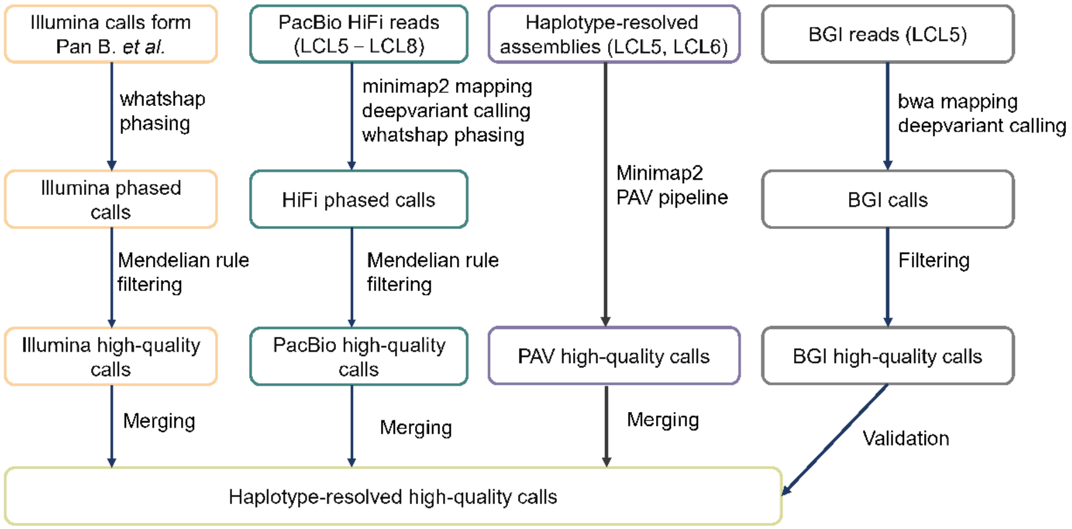

Fig. S11 SNV and indel detection and validation pipelines.

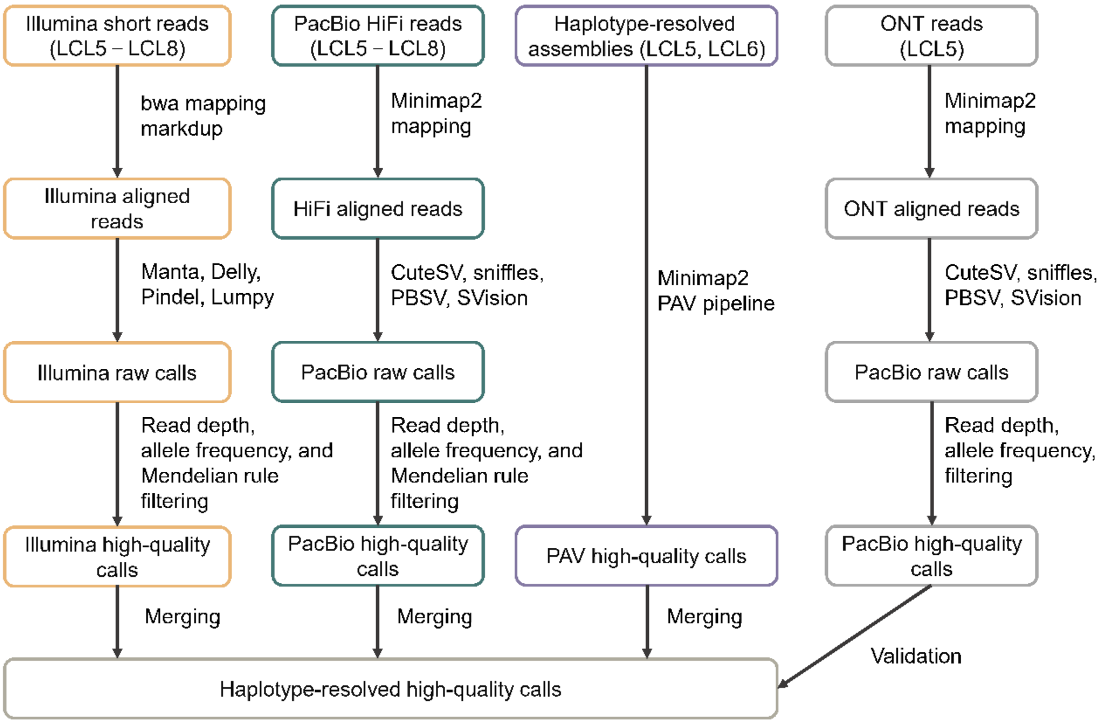

Fig. S12 Structural variant detection and validation pipelines

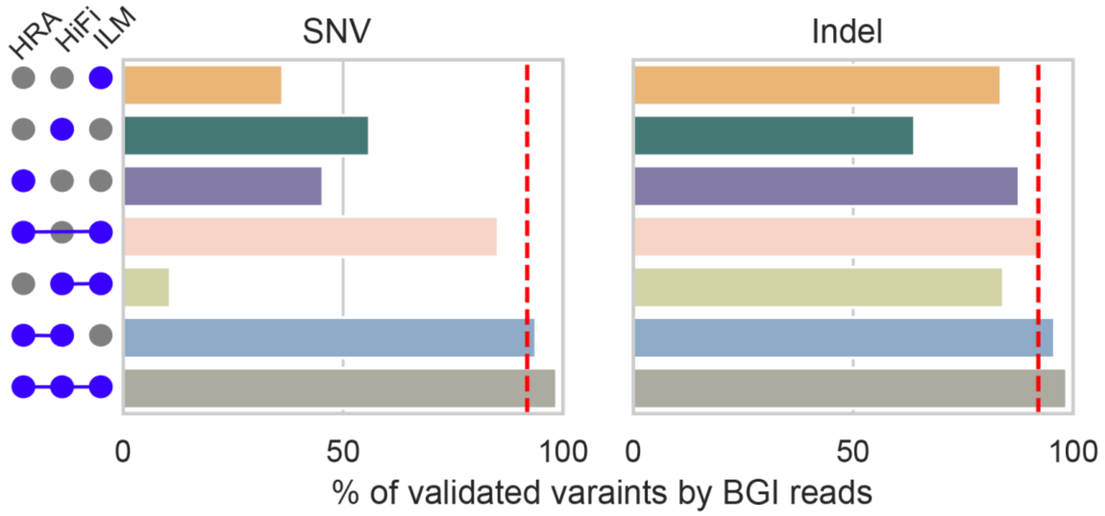

Fig. S13 Validated percentage of SNVs and indels across seven different combinations of three technologies. The red dotted lines represent the percentage of SNVs and indels that span repeat regions across the entire benchmarking set.

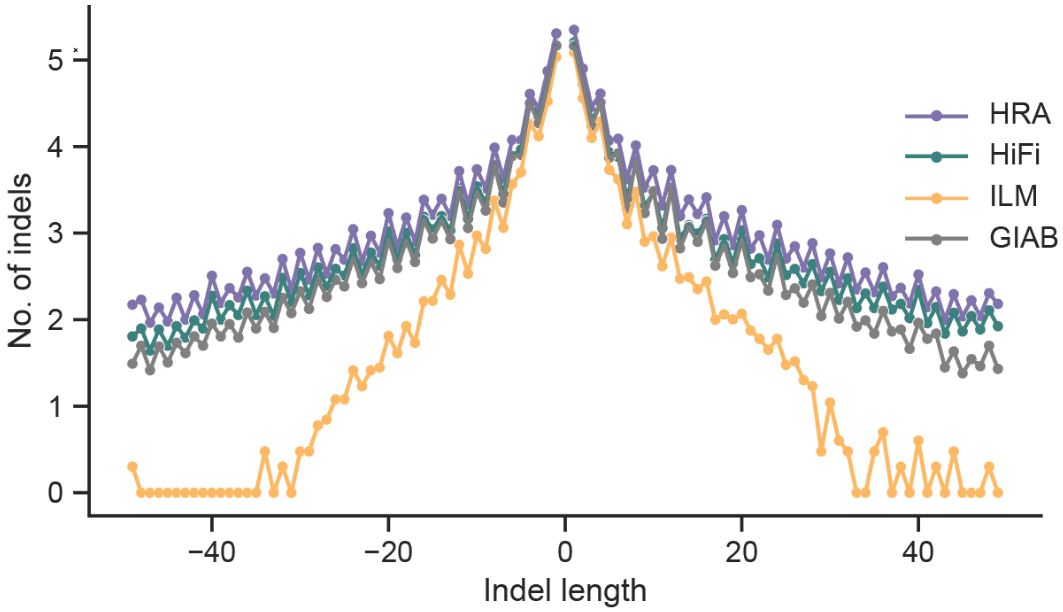

Fig. S14 Indel length distributions of HG002 and three technologies calls of Chinese Quartet twin daughters

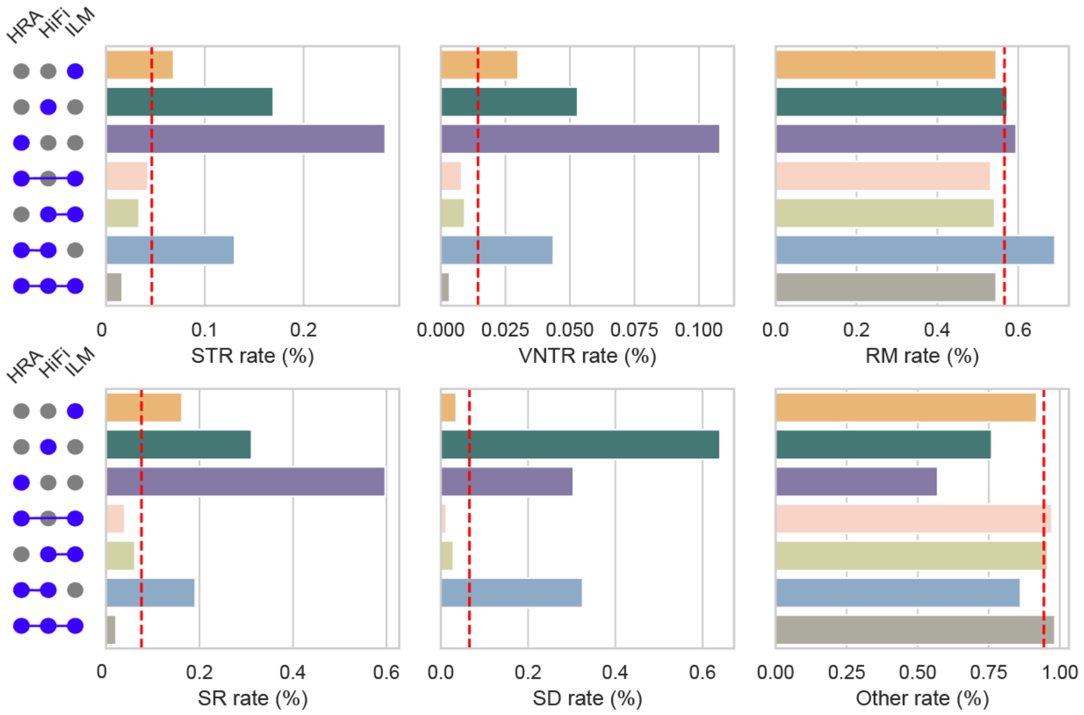

Fig. S15 SNV rate in repeat regions across different combinations of three technologies. The red dotted lines represent the percentage of SNVs that span the repeat regions across the entire benchmarking set. SD, segmental duplication; SR, simple repeat; VNTR, variable number tandem repeat; STR, short tandem repeat; RM, repeat mask regions; Other, regions excluding SD, SR, VNTR, STR, and RM.

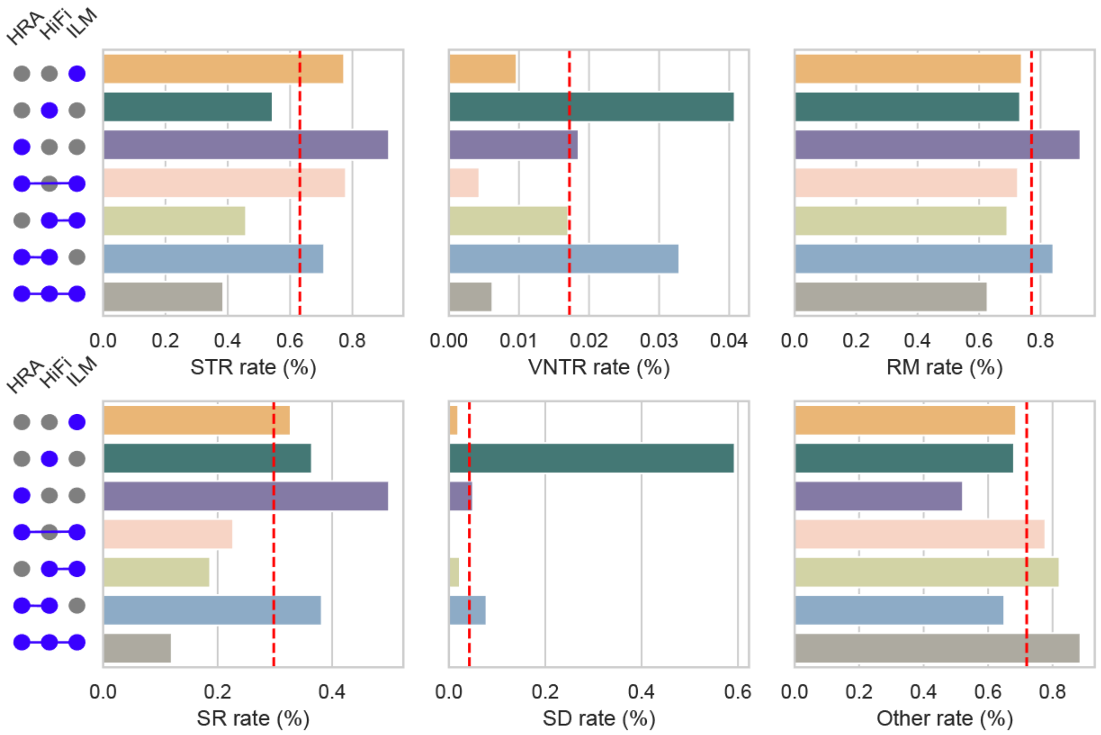

Fig. S16 Indel rate in repeat regions across different combinations of three technologies. The red dotted lines represent the percentage of indels that span the repeat regions across the entire benchmarking set. SD, segmental duplication; SR, simple repeat; VNTR, variable number tandem repeat; STR, short tandem repeat; RM, repeat mask regions; Other, regions excluding SD, SR, VNTR, STR, and RM.

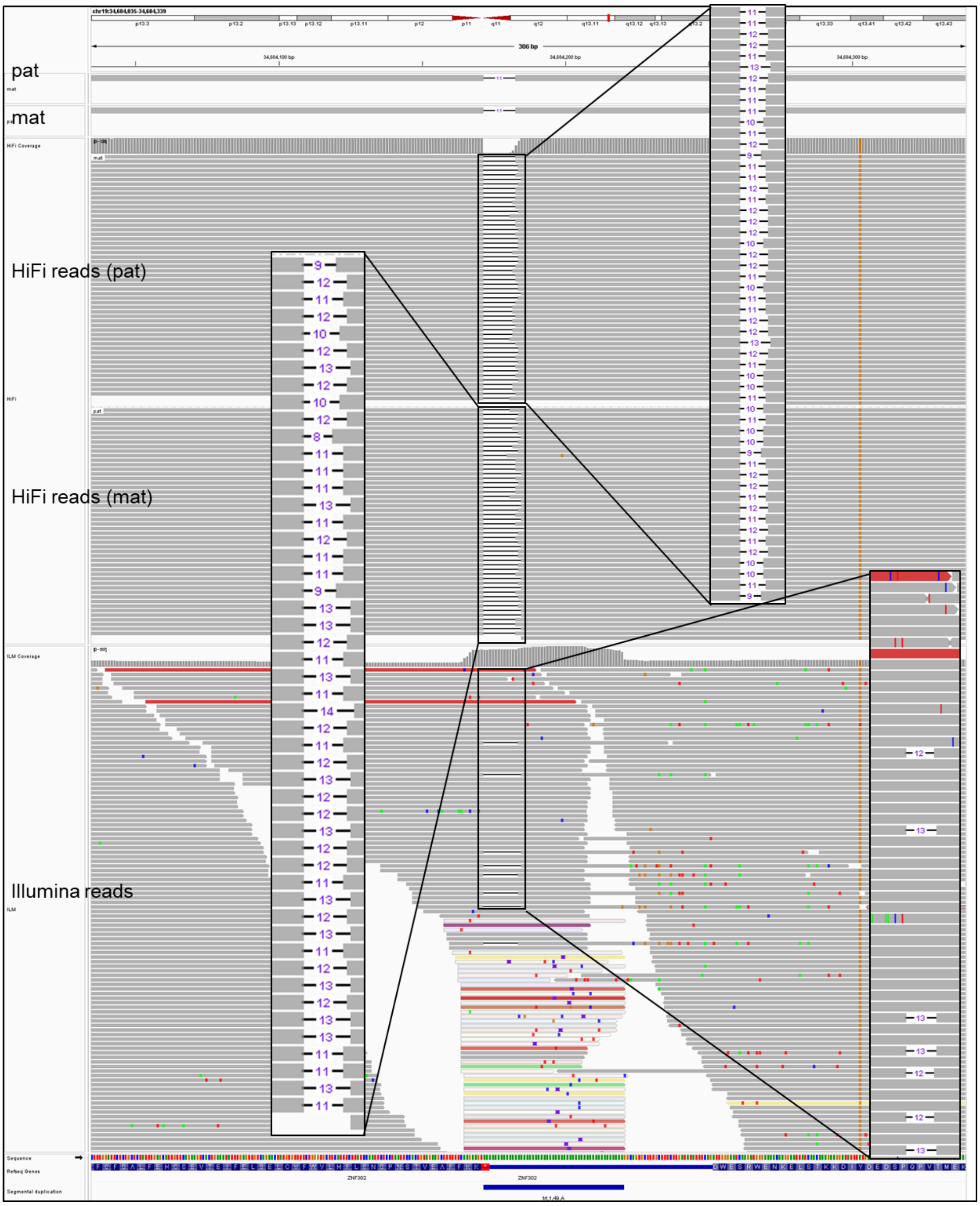

Fig. S17 IGV show the alignment of HRAs, HiFi reads, and Illumina reads to the reference genome (GRCh38) in a 49bp homopolymer region.

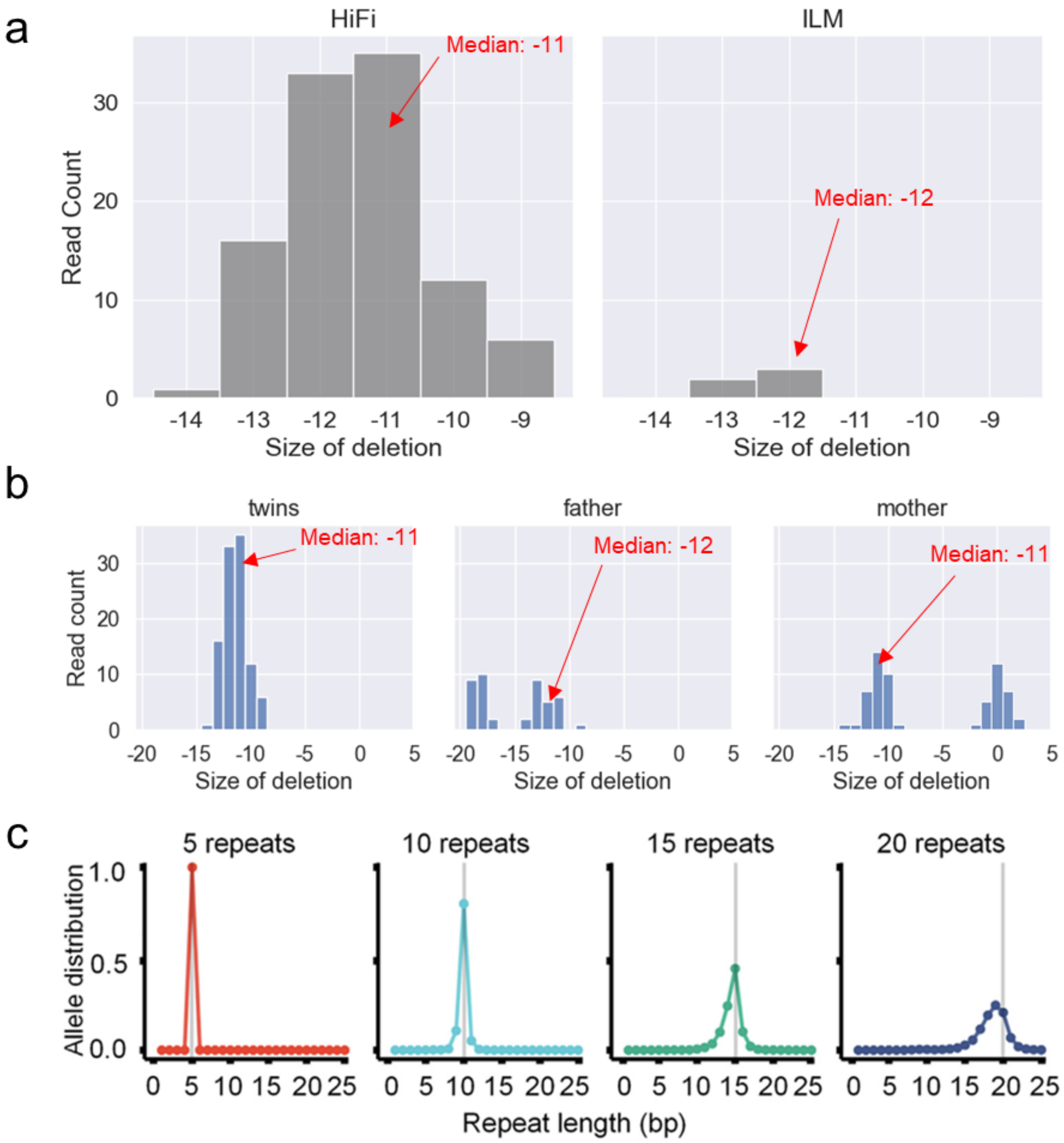

Fig. S18 Distribution analysis in homopolymer regions. a. The histogram shows the distribution of deletion size in the 49bp homopolymer regions by HiFi and Illumina PCR-free reads. b. The histogram shows the distribution of deletion size in the 49bp homopolymer regions based on HiFi reads from the twins and their parents. c. (From 10.1016/j.gpb.2020.02.001). Repeat length distribution of Illumina reads in homopolymers. The distributions flattened (the variances became larger and the modes deviated from expectation) with increases in the repeat length of microsatellites in the reference genome.

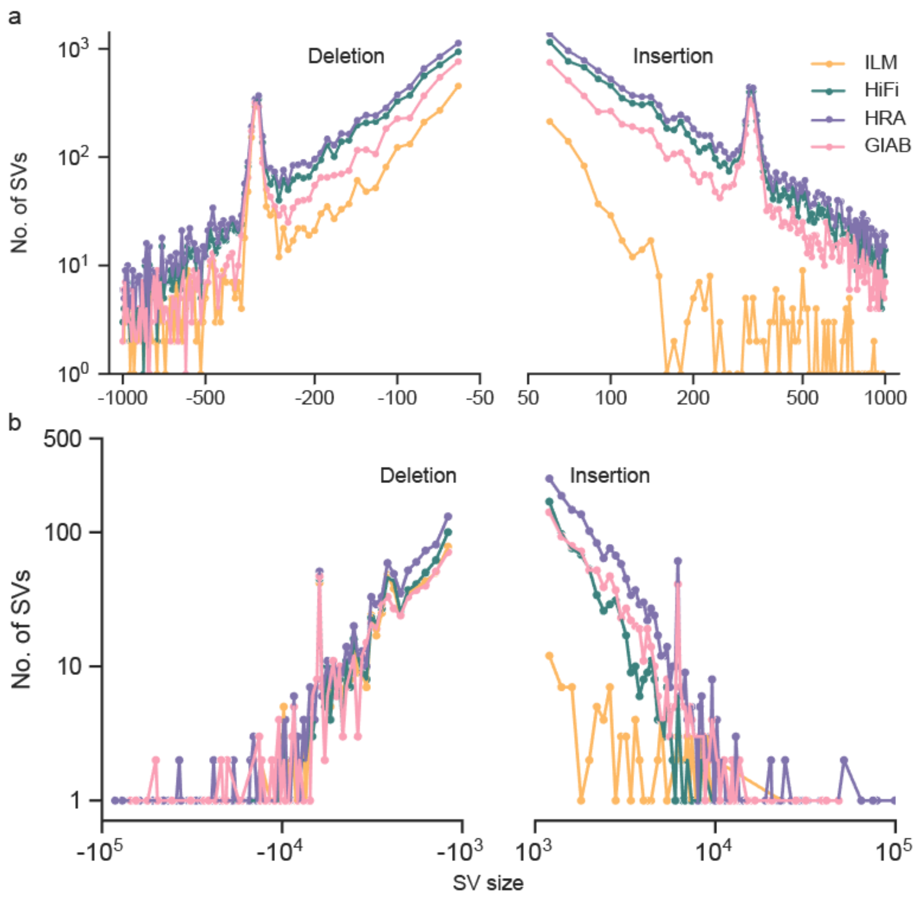

Fig. S19 SV length distributions of HG002 and three callsets of Chinese Quartet twin daughters.

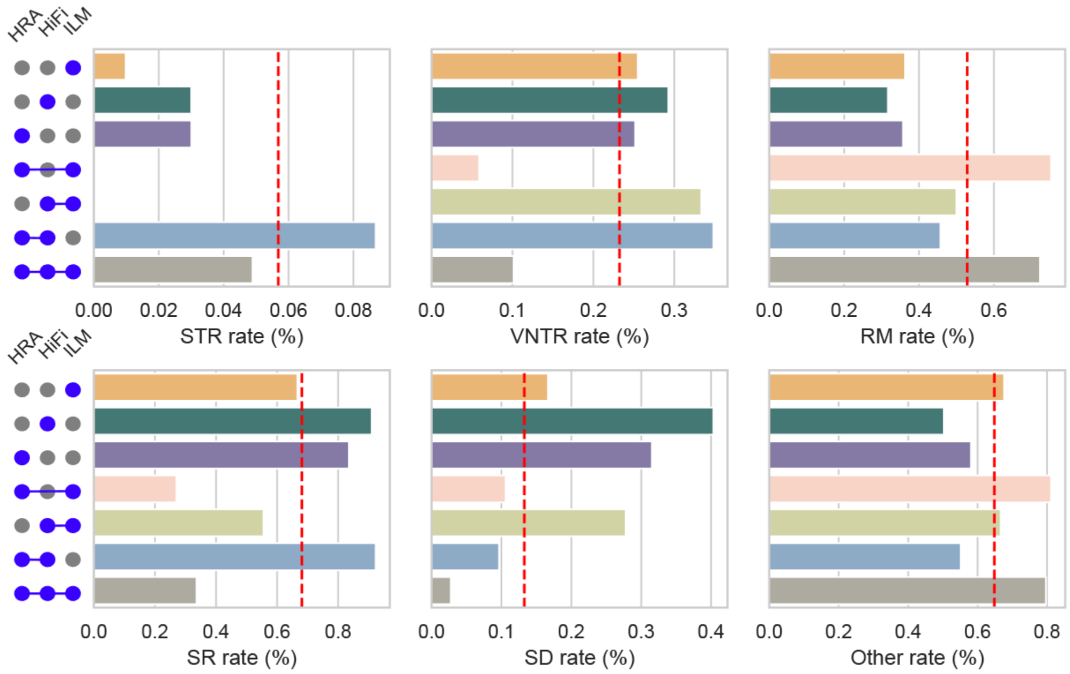

Fig. S20 Large deletion rate in repeat regions across different combinations of three technologies. The red dotted lines represent the percentage of deletions (⩾50bp) that span the repeat regions across the entire benchmarking set. SD, segmental duplication; SR, simple repeat; VNTR, variable number tandem repeat; STR, short tandem repeat; RM, repeat mask regions; Other, regions excluding SD, SR, VNTR, STR, and RM.

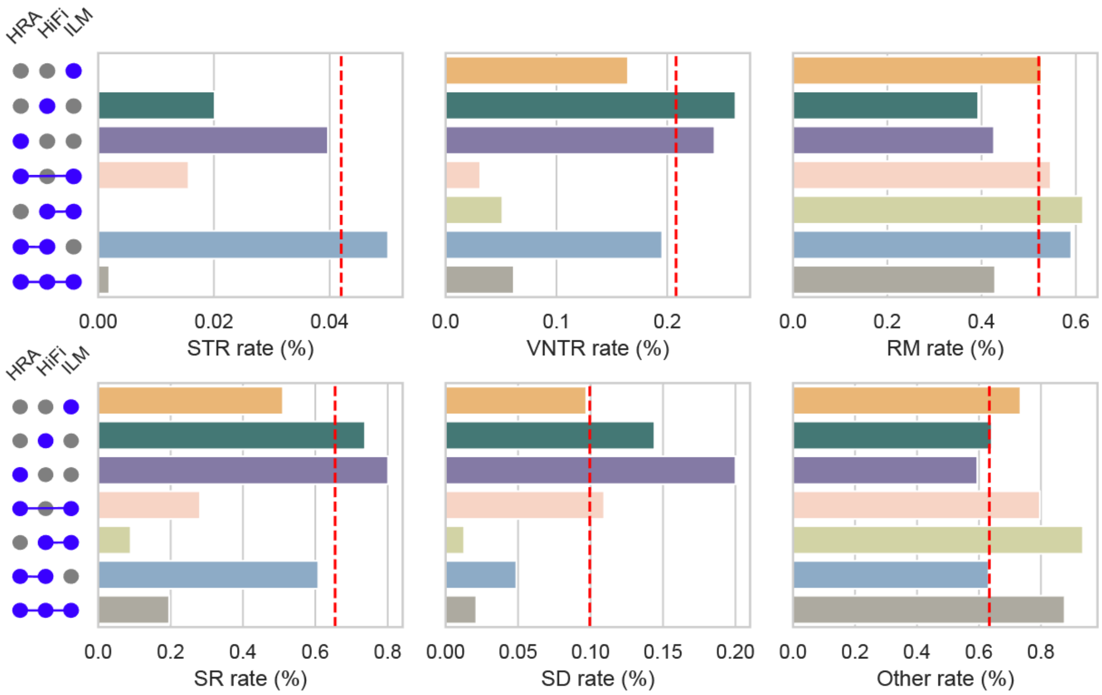

Fig. S21 Large insertion rate in repeat regions across different combinations of three technologies. The red dotted lines represent the percentage of insertions (≥50bp) that span the repeat regions across the entire benchmarking set. SD, segmental duplication; SR, simple repeat; VNTR, variable number tandem repeat; STR, short tandem repeat; RM, repeat mask regions; Other, regions excluding SD, SR, VNTR, STR, and RM.

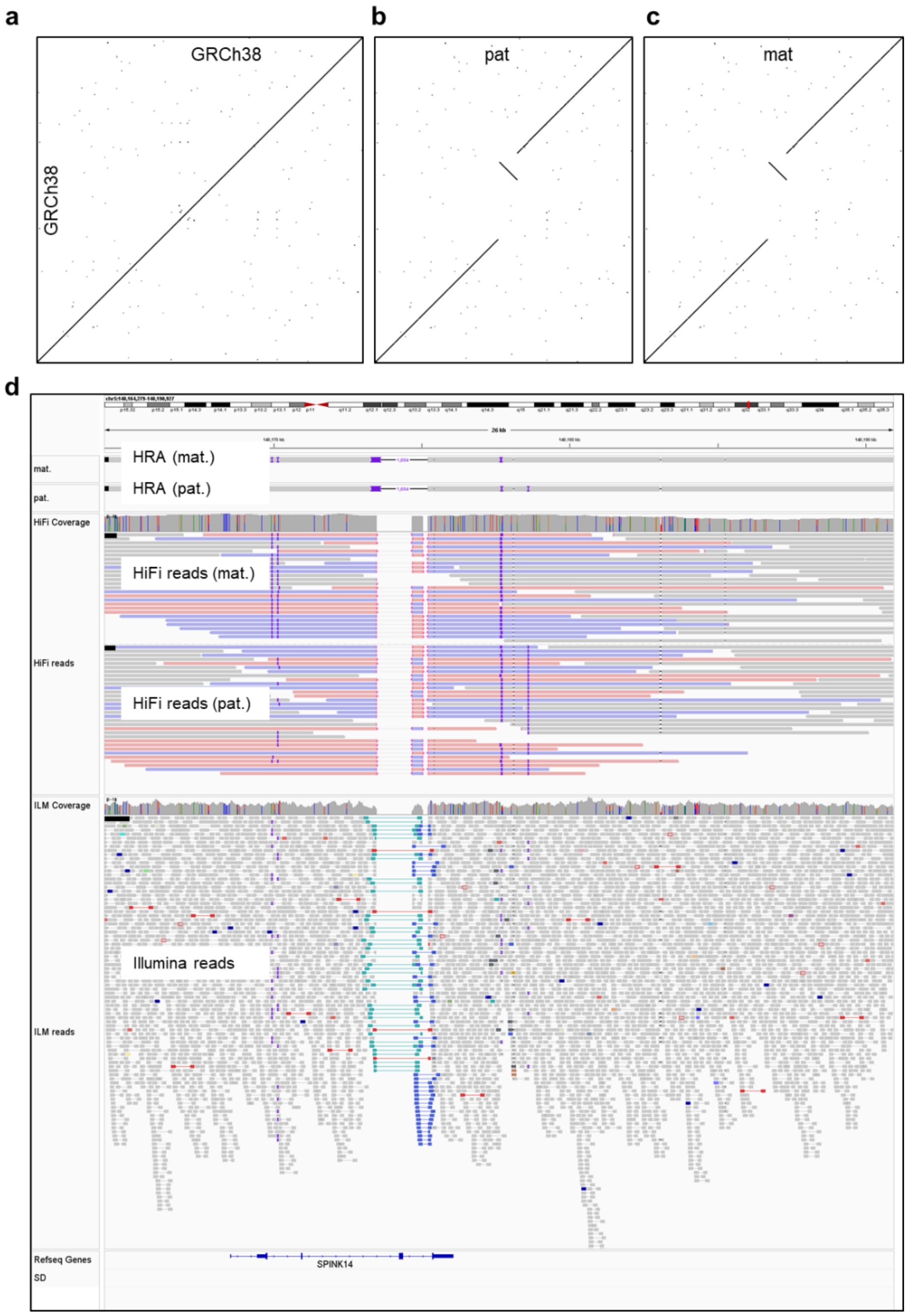

Fig. S22 True CSV (DEL+INV) example at chr5:148,170,966-148,177,603 a-c, Dotplots show the comparisons of sequence between GRCh38 and GRCh38, GRCh38 and paternal haplotype, as well as GRCh38 and maternal haplotype at this locus. d, IGV snapshot shows the alignments of haplotype-resolved assemblies, HiFi reads, and Illumina reads at this locus.

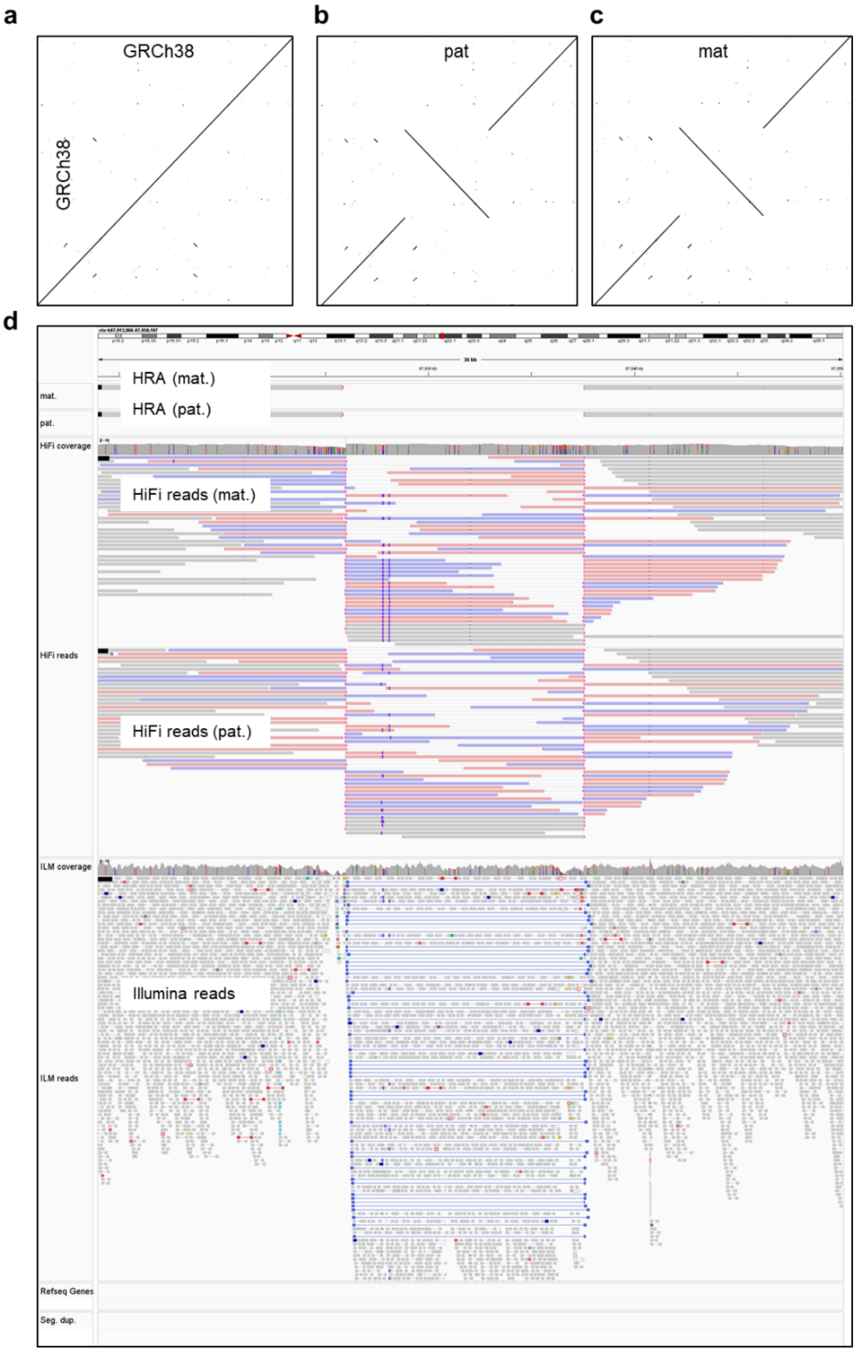

Fig. S23 True inversion example at chr4:87,913,966-87,950,107. **a-c,** Dotplots show the comparisons of sequence between GRCh38 and GRCh38, GRCh38 and paternal haplotype, as well as GRCh38 and maternal haplotype at this locus. **d,** IGV snapshot shows the alignments of haplotype-resolved assemblies, HiFi reads, and Illumina reads at this locus.

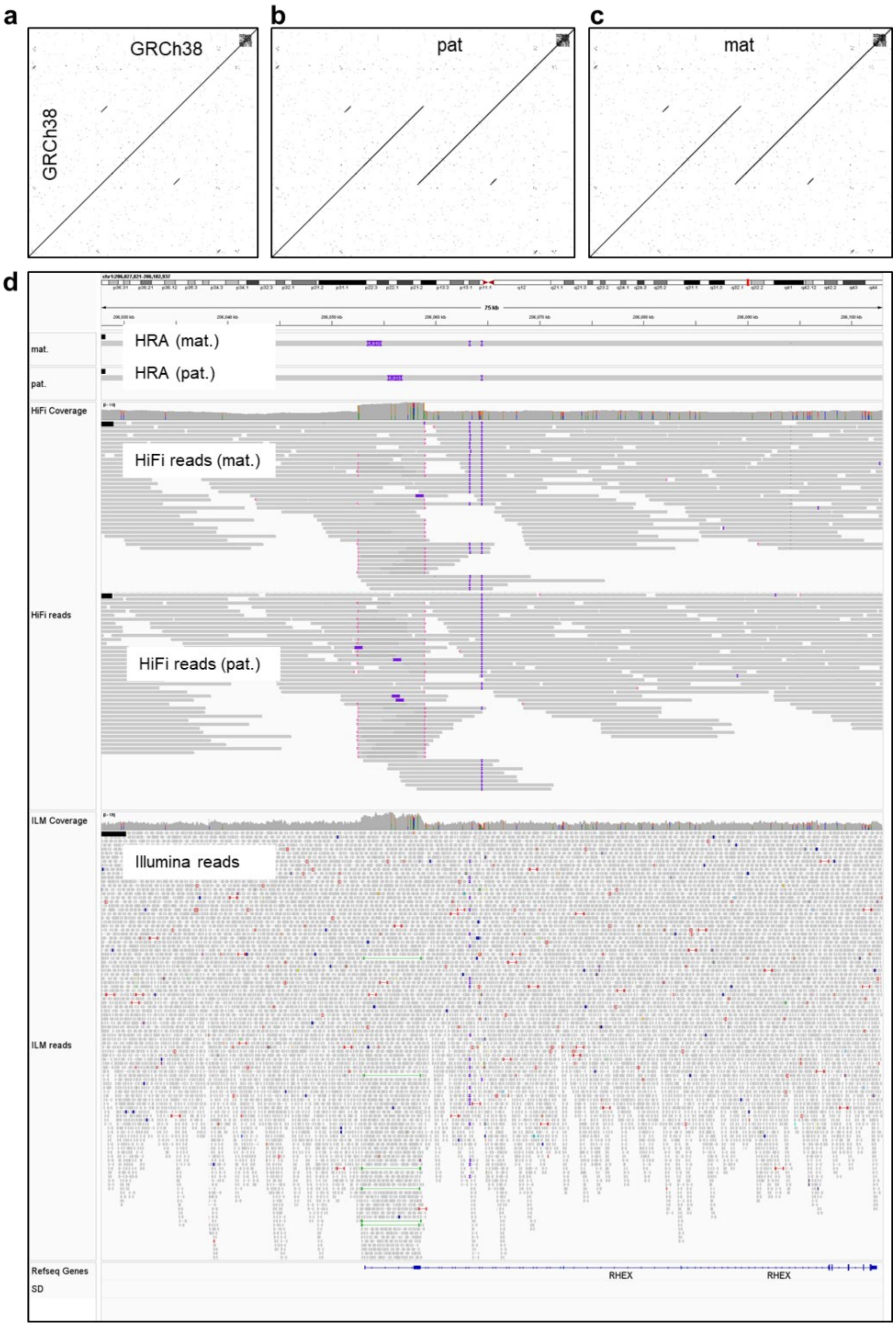

Fig. S24 Tandem duplication were reported as CSV at chr1:206,046,625-206,065,379. **a-c**, Dotplots show the comparisons of sequence between GRCh38 and GRCh38, GRCh38 and paternal haplotype, as well as GRCh38 and maternal haplotype at this locus. **d**, IGV snapshot shows the alignments of haplotype-resolved assemblies, HiFi reads, and Illumina reads at this locus.

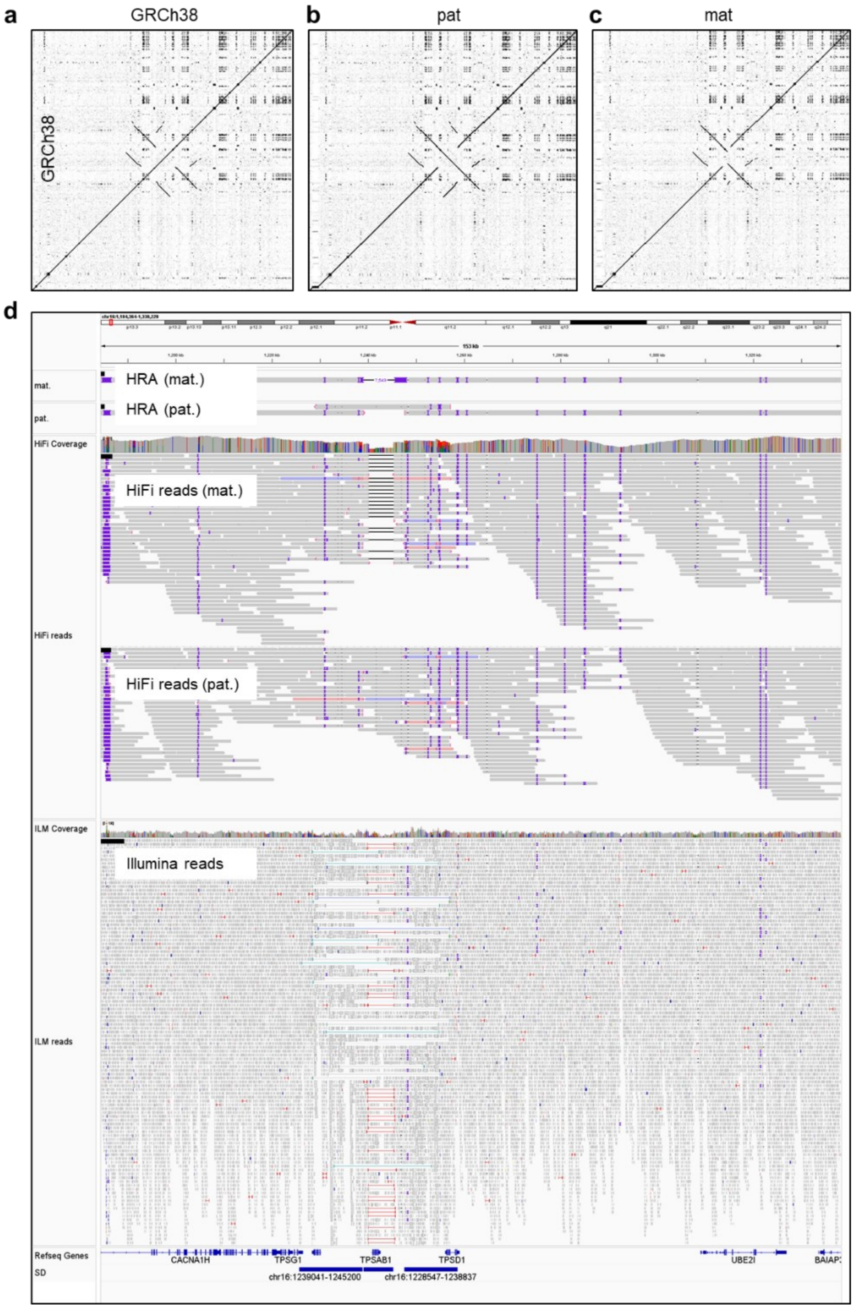

Fig. S25 Unsure CSV in repeat regions at chr16:1,222,853, 1,261,292. **a-c**, Dotplots show the comparisons of sequence between GRCh38 and GRCh38, GRCh38 and paternal haplotype, as well as GRCh38 and maternal haplotype at this locus. **d**, IGV snapshot shows the alignments of haplotype-resolved assemblies, HiFi reads, and Illumina reads at this locus.

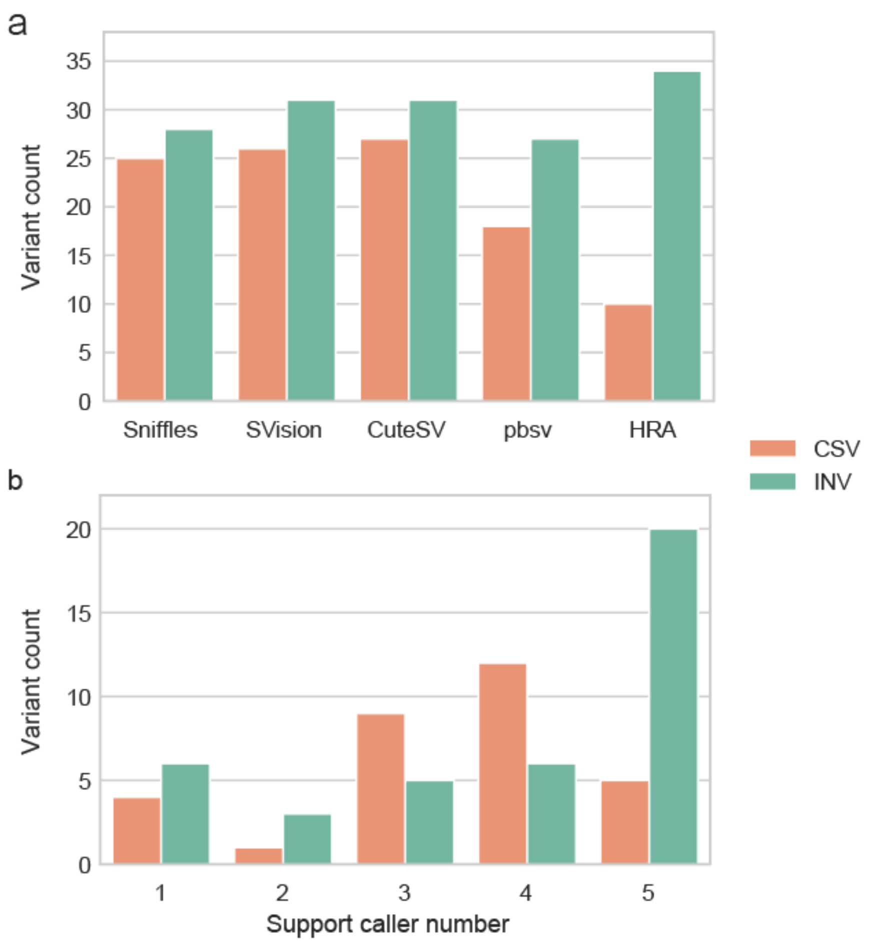

Fig. S26 Complex SVs and inversions in benchmarks. **a,** Bar plot shows the number of variants discovered by different callers. **b,** Bar plot shows the variant numbers of different supported callers.

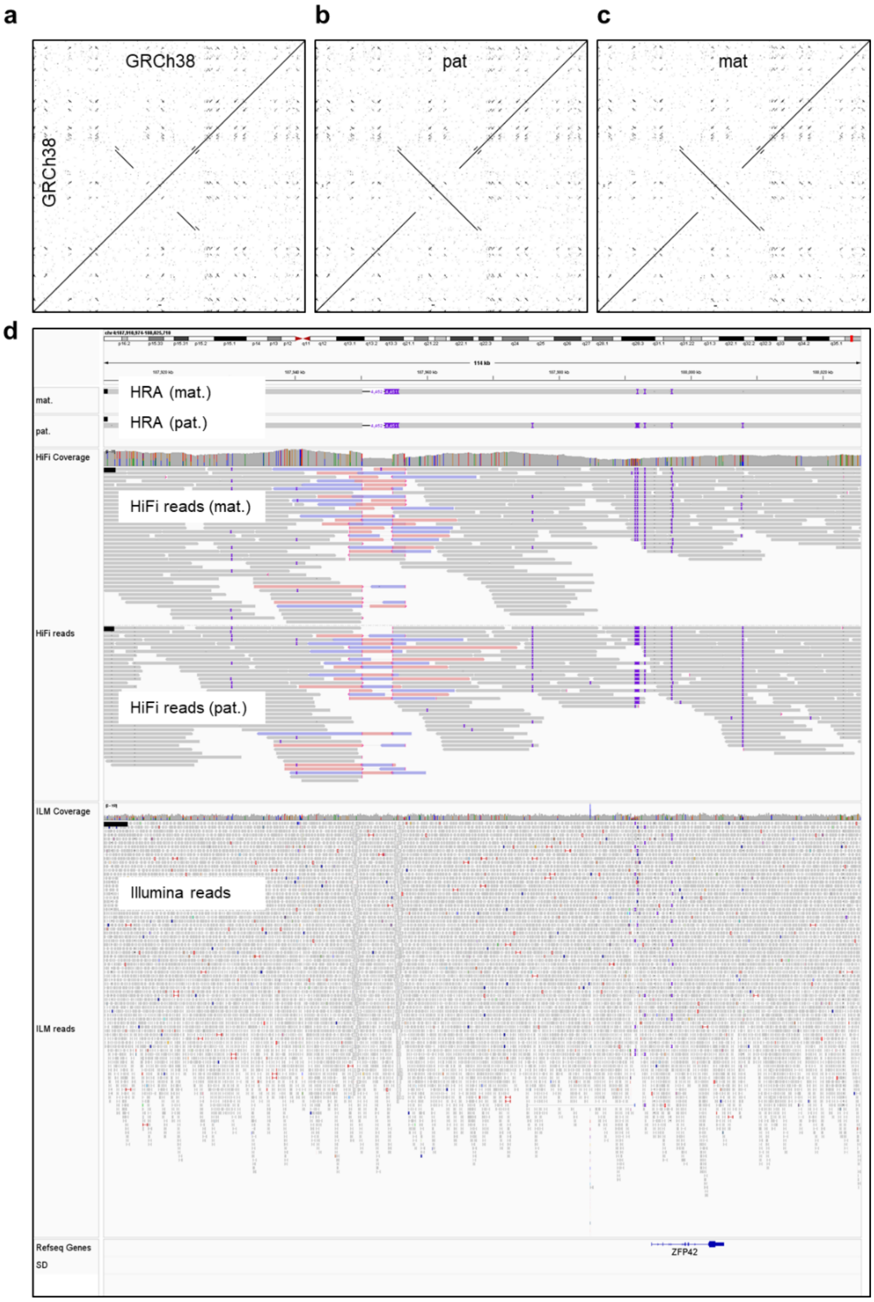

Fig. S27 Recurrent inversion at chr7:40,838,845-40,841,845. **a-c**, Dotplots show the comparisons of sequence between GRCh38 and GRCh38, GRCh38 and paternal haplotype, as well as GRCh38 and maternal haplotype at this locus. **d**, IGV snapshot shows the alignments of haplotype-resolved assemblies, HiFi reads, and Illumina reads at this locus.

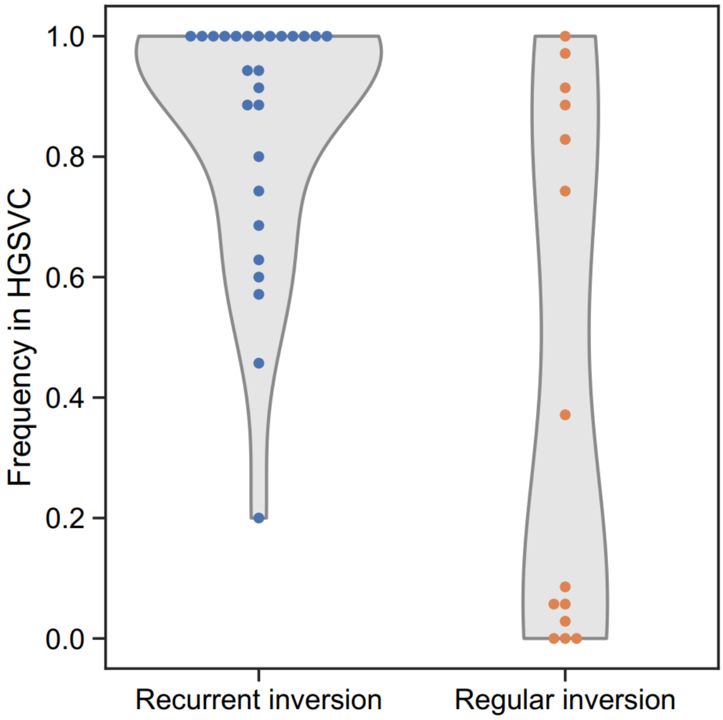

Fig. S28. Violin plot shows the frequency of inversion in the HGSVC dataset.

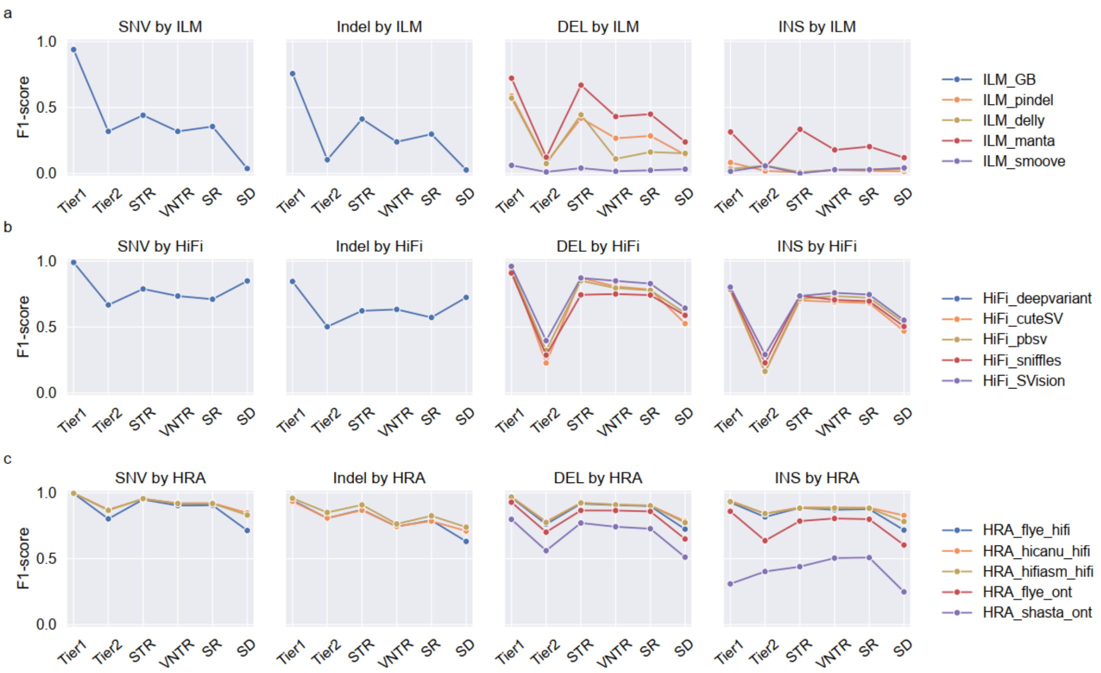

Fig. S29 F-scores of initial variants compared to the v2.0 benchmark of Chinese Quartet twin daughters. ILM_GB means the Illumina callset of the Chinese Quartet twin we obtain from the published paper (10.1186/s13059-021-02569-8).

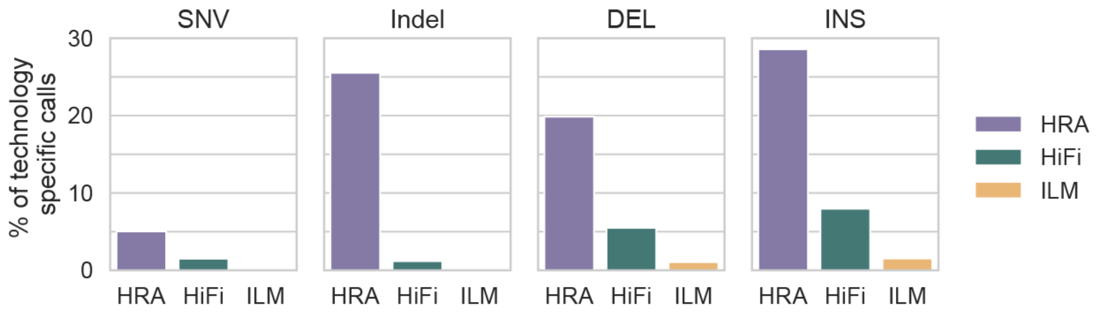

Fig. S30. Bar plots show the percentage of technology specific calls in v2.0 benchmark.

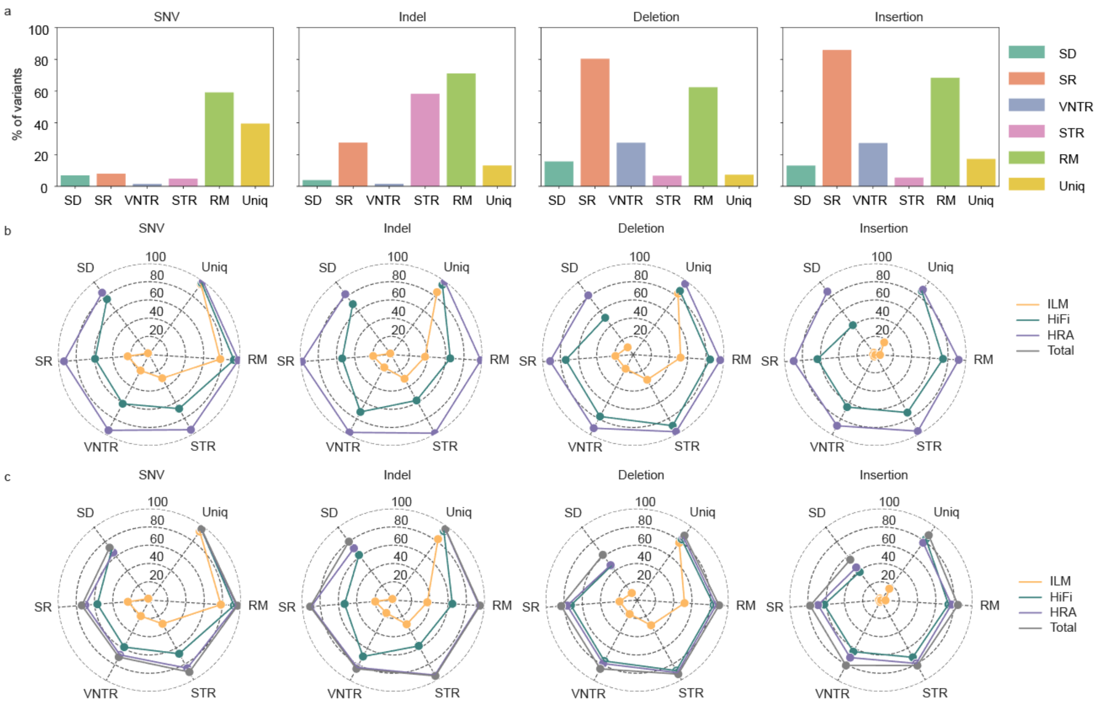

Fig. S31 Variant features in different types of regions**. a,** Percentage of variants in different types of regions. **b,** Radar plots show the percentage of SNVs, indels, large deletions, and insertions detected by ILM, HiFi, and HRA in distinct regions of the genome. **c,** Radar plots show the percentage of validated SNVs, indels, large deletions, and insertions in distinct regions of the genome. SD, segmental duplication; SR, simple repeat; VNTR, variable number tandem repeat; STR, short tandem repeat; RM, repeat mask regions; Other, regions excluding SD, SR, VNTR, STR, and RM.

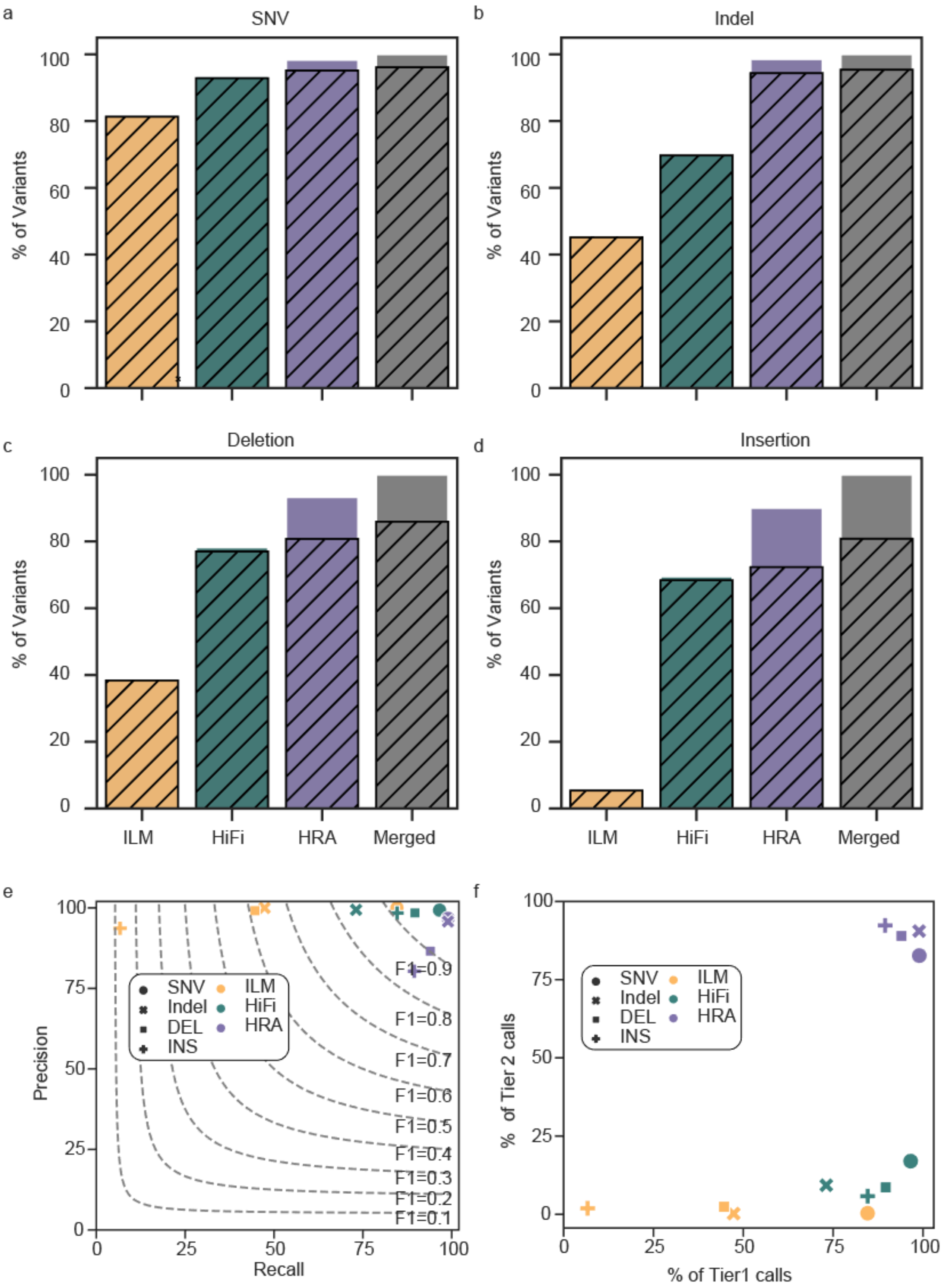

Fig. S32 Benchmark evaluation of Chinese Quartet twin daughters. **a – d,** Bar plot depicts the percentages of ILM, HiFi, and HRA calls in the v2.0 benchmarks, with gray stripes representing the validated percentages by BGI or ONT reads. **e,** Precision

and recall of ILM, HiFi, and HRA of the v2.0 Chinese Quartet benchmarks. Recall of each technology is represented by the ratio of high-confidence calls this technology detects to all validated calls. Precision of each technology is defined by the percentage of validated calls in all detected calls in this technology. The dashed line denotes the F1 score of the technology. **f,** Scatter plot shows the percentage of high-confidence (x-axis) and technology-specific (y-axis) calls across ILM, HiFi, and HRA.

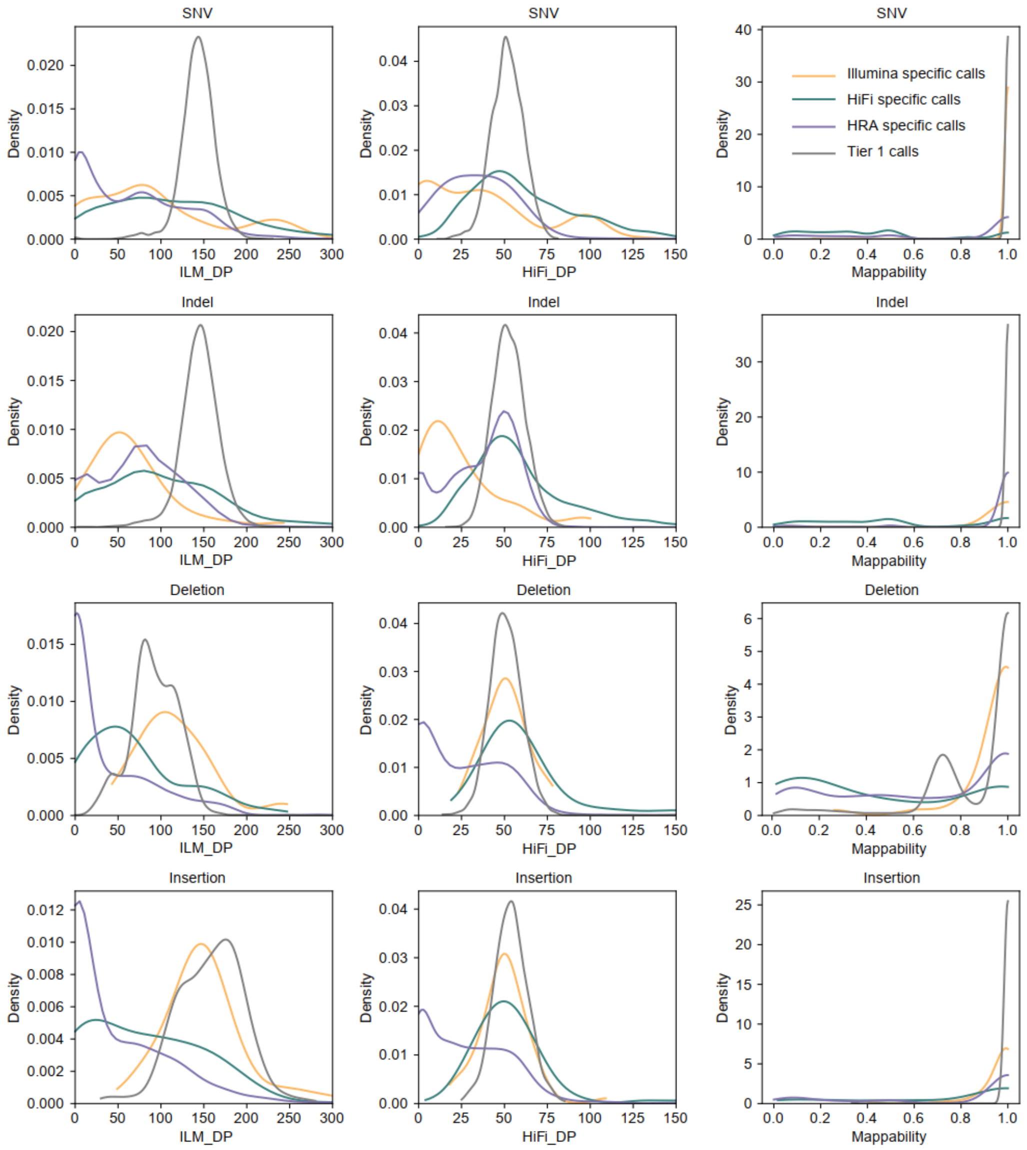

Fig. S33 The density plots show the difference in variant characteristics between high-confidence and technology-specific calls. Only reads with a mapping quality of at least 20 are used to compute read depths.

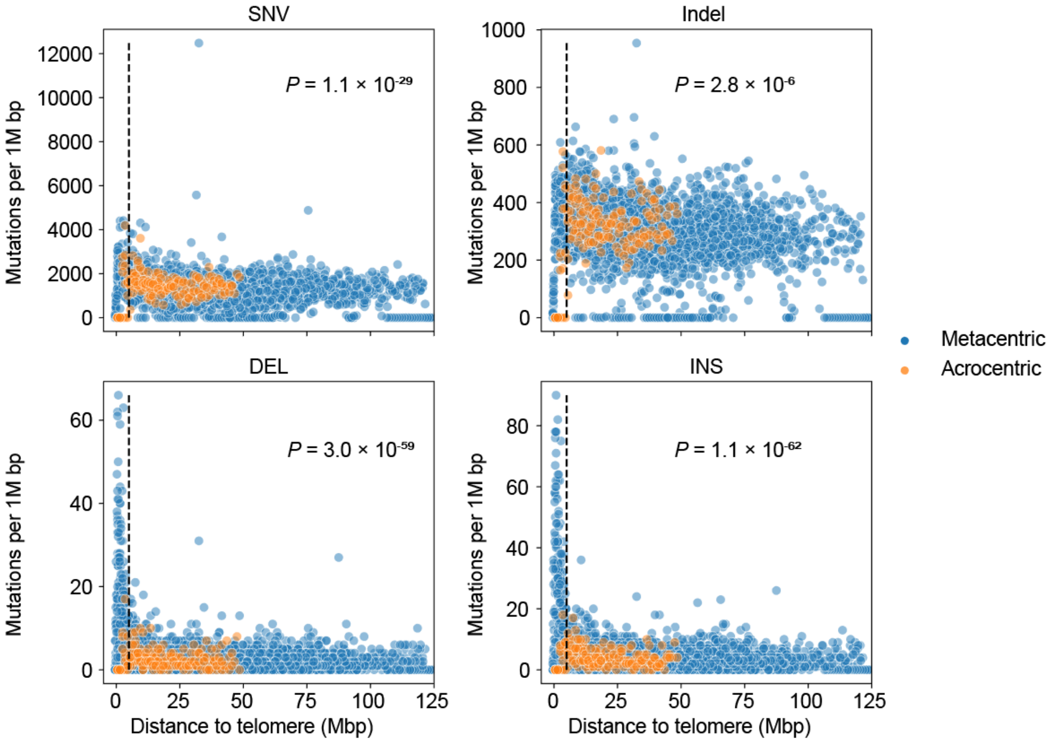

Fig. S34 Variant distribution of Chinese Quartet at the telomere. For each variant, the distance to the closest telomere of the chromosome is computed and divided into 1 Mbp bins. Variants are significantly enriched (Wilcoxon rank-sum one-sided test) within 5 Mbp of the telomere (dashed line left).

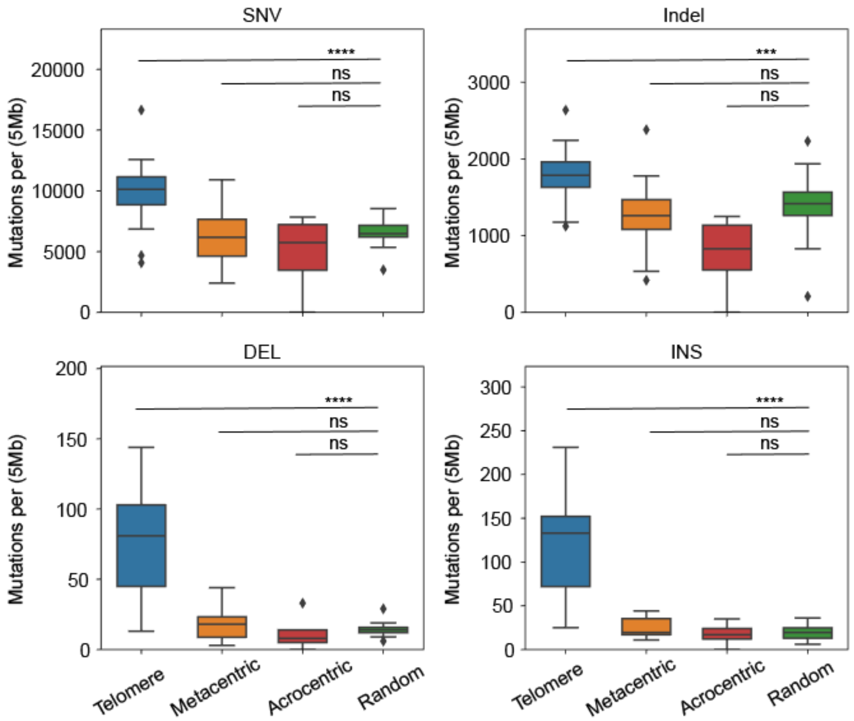

Fig. S35 Variant distribution of Chinese Quartet at telomere and centromere. The y-axis represents the number of variants located within 5 Mbp of the metacentric centromere, acrocentric centromere, and telomere. The number of variants located at telomeres is significant (Wilcoxon rank-sum one-sided test) more than other random background regions. ns, not significant. The box ranges from Q1 (the first quartile) to Q3 (the third quartile) of the distribution and the range represents the IQR (interquartile range). The median is indicated by a line across the box. The whiskers on box plots extend from Q1 and Q3 to 1.5×IQR. More extreme points are marked as outliers. *, $P < 0.05$; **, $P < 0.01$; ***, $P < 0.001$; ****, $P < 0.0001$.

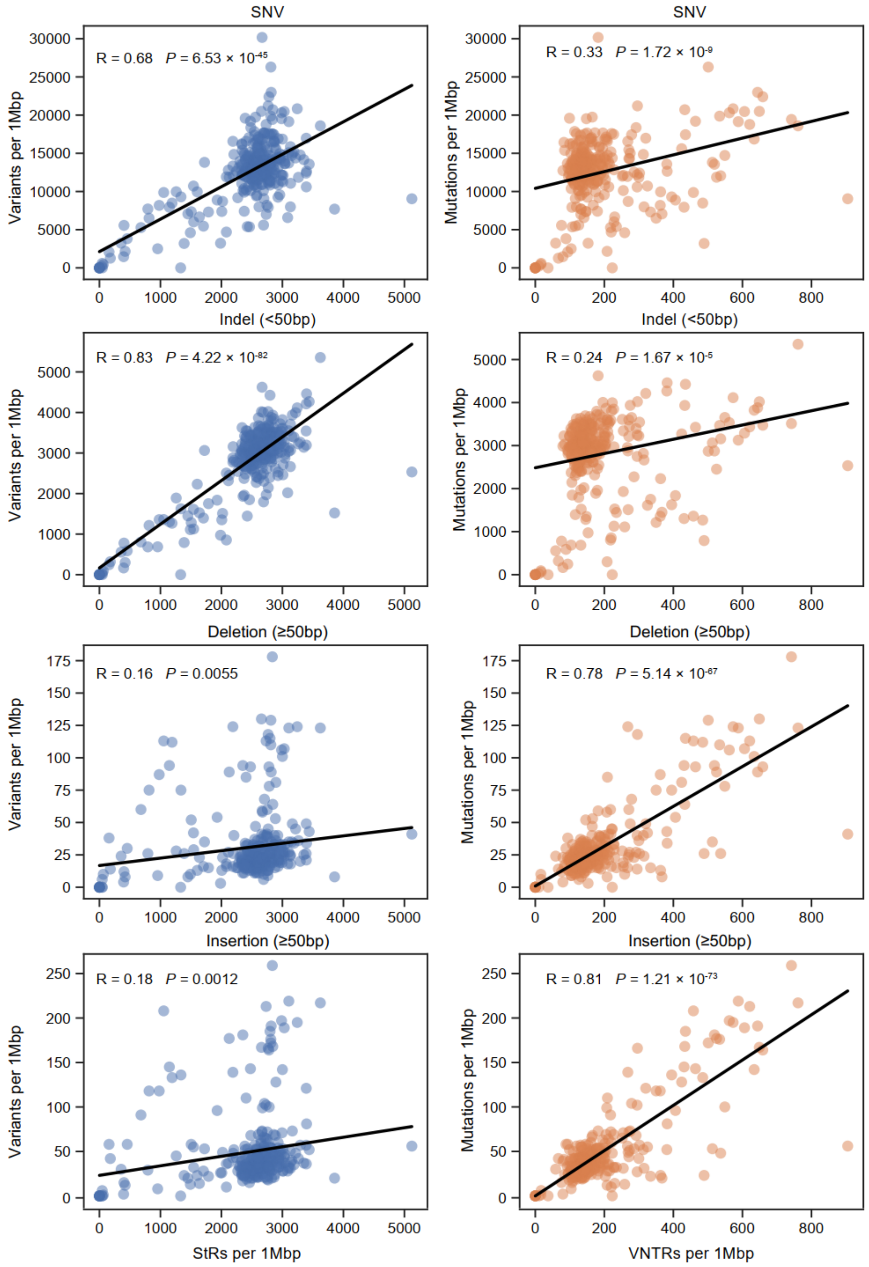

Fig. S36 STR/VNTR distribution and variant breakpoint correlation. The abundance of STRs is positively correlated with the distribution of SNVs (R = 0.73) and indels (R = 0.88), while VNTRs is positively correlated with structural variant (deletions, R= 0.82; insertion, R=0.85).

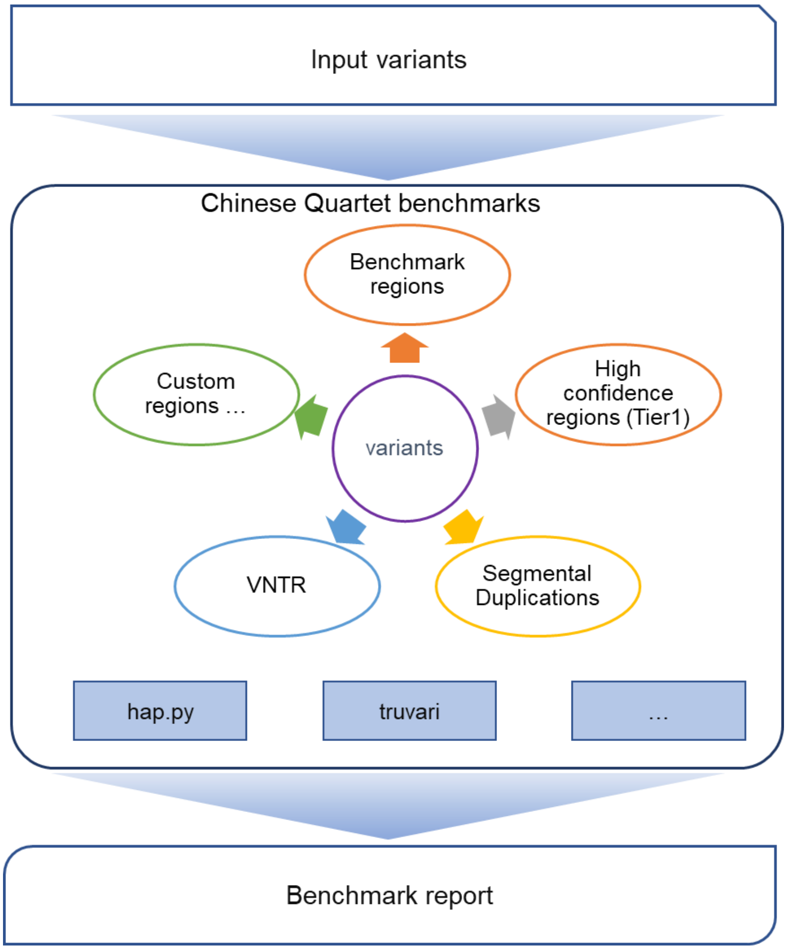

Fig. S37. Diagram for benchmark utility.

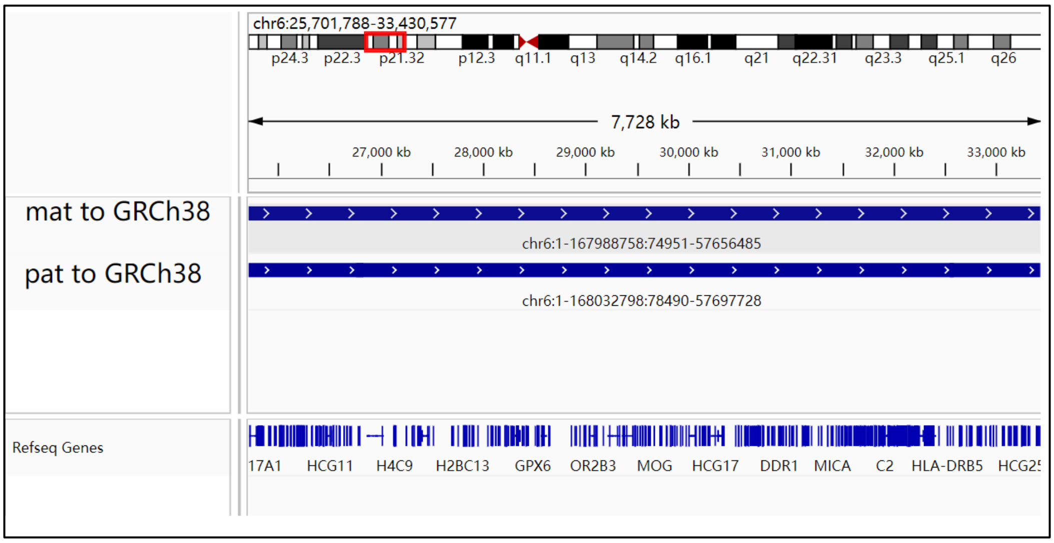

Fig. S38 IGV snapshot shows Chinese Quartet twins' assemblies to GRCH38 in xMHC regions.
