# Supplementary material for: Haplotype-resolved assemblies and variant benchmark of a Chinese Quartet
Source: Genome Biol. 2023 Dec 4;24:277. doi: 10.1186/s13059-023-03116-3 (PMC10694985; doi:10.1186/s13059-023-03116-3)
Supplement: Supplementary file 3 — Additional file 3: Supplementary notes. In this additional file, we provide detailed processes of data generation, genome assembly, and construction of variant benchmarks [100, 101]. [file 13059_2023_3116_MOESM3_ESM.pdf]

Supplementary Notes for

Haplotype-resolved assemblies and variant benchmark of a Chinese Quartet

Contents

1 Generation of sequencing data 1

1.1 Library preparation and sequencing of HiFi reads 1

1.2 Library preparation and sequencing of regular ONT reads 1

1.3 Library preparation and sequencing of ultra-long ONT reads 2

2 Evaluation of read phasing methods 2

2.1 Reference-based read phasing 2

2.2 Performance evaluation 3

3 Sequence assembly 5

3.1 Assembly parameters 5

3.2 Evaluation of complex regions 6

3.3 Gap filling and genome polishing of Chinese Quartet genome 6

4 Variant calling and the creation of variant benchmarking set 8

4.1 Variant filtering with Mendelian rule 8

4.2 Small variant detection 9

4.2.1 Small variant detection using Illumina reads 9

4.2.2 Small variant detection using HiFi reads 9

4.2.3 Small variant detection using haplotype-resolved assemblies 9

4.3 Structural variant detection 10

4.3.1 Structural variant detection by Illumina reads 10

4.3.2 Structural variant detection by HiFi reads 11

4.3.3 Structural variant detection by haplotype-resolved assemblies 11

4.4 Complex variant and inversion detection 12

4.5 Detection of *de novo* and putative somatic mutations 12

4.6 Benchmarking regions and high-confidence regions definition 13

4.7 Phasing regions definition 15

5 Variant benchmark evaluation 16

1 Generation of sequencing data

1.1 Library preparation and sequencing of HiFi reads

SMRTbell target size libraries were constructed for sequencing according to PacBio's standard protocol (Pacific Biosciences, CA, USA) using 15kb preparation solutions. The main steps for library preparation are: (1) gDNA shearing, (2) DNA damage repair, end repair and A-tailing, (3) ligation with hairpin adapters from the SMRTbell Express Template Prep Kit 2.0 (Pacific Biosciences), (4) nuclease treatment of SMRTbell library with SMRTbell Enzyme Cleanup Kit, (5) size selection, and (6) binding to polymerase. Briefly, a total amount of 15 μg DNA per sample was used for the DNA library preparations. The genomic DNA sample was sheared by gTUBEs (Covaris, USA) according to the expected size of the fragments for the library Single-strand overhangs were then removed, and DNA fragments were damage repaired, end repaired and A-tailed. The fragments were then ligated with the hairpin adaptor for PacBio sequencing. The library was then treated with nuclease using SMRTbell Enzyme Cleanup Kit and purified using AMPure PB beads. Target fragments were screened by BluePippin (Sage Science, USA) with the Agilent 2100 Bioanalyzer (Agilent technologies, USA) used to detect the size of library fragments. Sequencing was performed on a PacBio Sequel II instrument with Sequencing Primer V2 and Sequel II Binding Kit 2.0 at the Genome Centre of Grandomics (Wuhan, China).

1.2 Library preparation and sequencing of regular ONT reads

A total amount of 3-4 μg DNA per sample was used as input material for the ONT library preparations. After the sample was qualified, size-selection of long DNA fragments was performed using the Pippin HT system (Sage Science, USA). The ends of the DNA fragments were then repaired, and A-ligation reactions were conducted with the NEB Next Ultra II End Repair/dA-tailing Kit (Cat# E7546). The adapter in the SQK-LSK109 kit (Oxford Nanopore Technologies, UK) was used for further ligation

reactions. The DNA library was quantified using a Qubit® 4.0 Fluorometer (Invitrogen, USA). Approximately 700ng DNA libraries were constructed and sequenced using a Nanopore PromethION (Oxford Nanopore Technologies, UK) at the Genome Centre of GrandOmics (Wuhan, China).

1.3 Library preparation and sequencing of ultra-long ONT reads

For each ultra-long Nanopore library, approximately 8-10 μg of gDNA was size selected (>50 kb) with the SageHLS HMW library system (Sage Science, USA), and processed using the Ligation Sequencing 1D kit (SQK-LSK109, Oxford Nanopore Technologies, UK) according to the manufacturer' s instructions. Approximately 800ng DNA libraries were constructed and sequenced using a the PromethION (Oxford Nanopore Technologies, UK) at the Genome Centre of GrandOmics (Wuhan, China).

2 Evaluation of read phasing methods

2.1 Reference-based read phasing

We firstly obtained a high-confidence set of 3,249,650 single nucleotide variants (SNVs) and 404,882 indels of the family from a previous study [11]. The variants of the monozygotic twin daughters were phased using the "phase" command of whatshap [44] (v1.1) with parent-child information and each child's HiFi reads. The parameters were as follows:

```
whatshap phase --indels -r <GRCh38ref.fa> --ped <ChineseQuartet.ped> -o <output.phased.vcf> <input.vcf> <input.children.bam>
```

We then aligned the HiFi, ONT, and ultra-long ONT reads from each twin to GRCh38 using minimap2 [60] (v2.20-r1061) with recommended parameters at github (https://github.com/lh3/minimap2).We added haplotype tags to the BAM files using the "haplotag" command of whatshap, and assigned reads with haplotype tags to their respective haplotypes using the "split" command of whatshap. The parameters were as

follows:

whatshap haplotag -o <output.bam> -r <input.ref.fa> --sample <sample_name> --output-haplotag-list <output.sample.list> < output.phased.vcf > <input.bam>

whatshap split --only-largest-block --read-lengths-histogram <output.read_len> --output-h1 <output.pat.bam> --output-h2 <output.mat.bam> --output-untagged <output.untag.bam> <output.bam> < output.sample.list>

samtools fastq -0 <output.pat.fq/output.mat.fq/output.untag.fq> -@ <threads> <output.pat.bam/output.mat.bam / output.untag.bam>

Reads which were either unassigned to a haplotype or unmapped to GRCh38 were randomly assigned to the two haplotypes with a custom script (https://github.com/xjtu-omics/ChineseQuartetGenome/blob/main/pipelines/read_phasing/read_phase.smk) . For each sequencing technology, the proportion of reads which were phased is given in Supplementary Table S2.

2.2 Performance evaluation

We used the reference-based approach (described in Section 2.1) to split the children's reads into two haplotypes according to their parents' reads. To compare the performance in this step with a reference-free phasing method, we also split the reads with canu [37] (v2.2) using the commands: "canu useGrid=false -haplotype -p output -d genomeSize=3.1g -haplotypePaternal father.fq.gz -haplotypeMaternal mother.fq.gz -pacbio-hifi child2.fq.ga child2.fq.gz". We found that our reference-based approach could phase 76.2% of the HiFi reads, 64.4% of the regular ONT reads, and 70.5% of the ultra-long ONT reads, while canu could phase 65.3% of the HiFi reads, 79.9% of the regular ONT reads, and 83.0% of the ultra-long ONT reads (Supplementary Table S2).

To further evaluate the phasing accuracy, we also split the unphased reads into two haplotypes randomly. We then used the GenomeScope [100] pipeline to evaluate the

heterozygosity rate of each haplotype, considering a lower heterozygosity rate to indicate a higher phasing accuracy. The steps taken and parameters were:

jellyfish count -C -m 23 -s 2G -t 24 -o output.js

jellyfish histo -t 24 output.js > output.hist

Rscript genomescope.R output.hist 23 10000 output_dir

Our reference-based phasing method achieved a phasing rate of 76.19% for HiFi reads, which is higher than the 65.34% achieved by canu. Moreover, our method demonstrated lower heterozygosity rates for paternal (0.0243-0.0251) and maternal (0.0235-0.0244) reads, whereas canu had higher heterozygosity rates for both paternal (0.3129-0.3185) and maternal (0.2819-0.2871) reads (Supplementary Table S2). These results indicate the superior phasing accuracy of our reference-based method. For ONT reads, although our method obtained a slightly lower phasing rate than canu, it also achieved higher phasing accuracy. In summary, under the promise of high phasing rate, our reference-based approach achieved a higher phasing rate. In summary, both our reference-based method and canu achieved comparable phasing rates, but our reference-based method achieved higher phasing accuracy.

Compared to reference-free phasing approaches, the reference-based method may be negatively affected by reads that cannot be mapped or that have low mapping quality. We found that 0.002% (569) of the total HiFi reads were unmapped to GRCh38, of which only 19 could be phased using canu. We also observed that 9.4% (2,223,420) of the total HiFi reads had a mapping quality < 20, of which 69.2% and 31.3% were phased using canu and our approach, respectively. We found that these low-mapping-quality reads spanned 106.0 Mbp regions of GRCh38, within which were found, in total, 343,359 SNVs (8.19%), 26,327 indels (3.02%), 1,103 large (≥ 50bp) deletions (10.40%), and 1,544 large (≥ 50bp) insertions (9.30%). However, only 31,932 of these SNVs (9.30%), 1,015 of these indels (3.86%), 170 of these large deletions (15.41%), and 108 of these large insertions (7.00%) remained unphased.

3 Sequence assembly

3.1 Assembly parameters

In this study, we merged the reads of the two twin samples for each haplotype and assembled each haplotype with four assemblers. Specifically, we assembled HiFi reads using hifiasm [38] (v0.15.5), hicanu [47] (v-r10117), and flye [46] (v2.8.3-b1695), and assembled ONT reads with flye [46] (v2.8.3-b1695) and shasta [45] (v0.7.0). The parameters of each assembler were as follows:

- Hifiasm for HiFi reads:

hifiasm -o <output> -t <threads> <input.fqs>

- hicanu for HiFi reads:

ulimit -Su 100000 && canu useGrid=false maxThreads=<threads>-p <assm_prefix> -d <assm_dir> genomeSize=3.1g -pacbio-hifi <input.fqs>

- flye for HiFi reads:

flye --pacbio-hifi <input.fqs> --genome-size 3.1g --out-dir <output> --threads <threads>

- flye for ONT reads:

flye --nano-raw <input.fqs> --genome-size 3.1g --out-dir <output> --threads <threads>

- shasta for ONT reads:

shasta --input <input.fqs> --threads <threads> --assemblyDirectory <output> --config <shasta_config> --memoryMode filesystem --memoryBacking 2M --command assemble

3.2 Evaluation of complex regions

Complex regions in the diploid human genome, such as segmental duplications, are challenging to validate through PCR or other biology experiments. Hence, we extracted the feature of HiFi read depth to evaluate the structure of the genome in these complex regions. We first aligned reads from paternal and maternal haplotypes to their corresponding genomes as well as to both GRCh38 and CHM13-T2T using minimap2 with default parameters. We considered that incorrect alignment in repetitive regions could affect the read depth, and that accordingly the read depth in these regions when aligned against CHM13-T2T could be used as a baseline. To detect abnormal read depths, we then randomly selected 1000 bins of 1000bp across each autosome, and defined the expected ranges of the read depth according to the interquartile range (IQR) of read depths in these bins. Depths lower than Q1–1.5*IQR or larger than Q3+1.5*IQR were defined as abnormal depths, where Q1 is the first quartile of the depth, Q3 is the third quartile, and IQR is Q3-Q1. The ratio of abnormal bins to total bin in the regions is calculated to evaluate the genome structure. For example, we found that the ratio of abnormal bins in our assemblies was comparable to CHM13-T2T in chr17:21,523,754-22371820, which suggested our genome achieved comparable quality with CHM13-T2T in this region (Supplementary Fig. 6). However, if we aligned the reads of our assemblies to GRCh38 and CHM13-T2T, the abnormal bins were greater those of the baseline.

3.3 Gap filling and genome polishing of Chinese Quartet genome

To improve the continuity of the assemblies, we scaffolded the hifiasm contigs using ragtag [71], guided by CHM13-T2T [31]. Next, we utilized the contigs produced by HiCanu, Flye, and shasta to fill in the gaps of the hifiasm scaffolds, which include three steps (https://github.com/PengJia6/gapless).

Firstly, the scaffold was split into 100bp k-mers, and only k-mers within 200kb

upstream and downstream of gaps were retained as candidate anchors. The candidate anchors were aligned to the scaffold using bwa mem (v0.7.17) and those k-mers with mapping quality more than 30 and without any mismatch were finally retained as anchors for next step.

Next, we aligned the anchors from the scaffold to the contigs to obtain potential gap sequences. We used bwa mem (v0.7.17) to align anchors to contigs, and anchors with mapping quality more than 30 were retained. We grouped the upstream and downstream anchors by contigs. For each gap, contigs with more than 10% upstream and downstream anchors of the scaffold were retained for gap filling. For each contig, its score for gap filling is calculated by the ratio of anchors in the contig to anchors of scaffolds.

Thirdly, we selected the contig with the greatest score to fill the gap. We selected the two closest anchors between upstream and downstream in a contig as representatives. The sequence between the two anchors in the contig was extracted to replace corresponding sequences in scaffold.

We then polished the genomes with NextPolish [49] (v1.3.1).The parameters of NextPolish were as follows:

rerun = 3s

parallel_jobs = 6

multithread_jobs = 24

genome_size = auto

lgs_options: -min_read_len 10k -max_read_len 150k -max_depth 120

lgs_minimap2_options = -x map-pb

4 Variant calling and the creation of variant benchmarking set

4.1 Variant filtering with Mendelian rule

In this study, we evaluated the Illumina and HiFi variant calls of the twins in the Chinese Quartet using Mendelian rules. Variants that followed Mendelian inheritance were included in the germline variant calls, while those that violated the rules were classified as error calls, *de novo* mutations, or somatic mutations.

The pseudo code of Mendelian rule filtering are as follows:

Procedure of Mendelian rule filtering

input: variants of the family, $V = \{v_1, v_2, \ldots v_i \ldots v_n\}$); where $v_{i=(}v_i^{d1}, v_i^{d2}, v_i^m, v_i^f)$, v_i^{d1}, v_i^{d2}, v_i^f, and v_i^m represented the ith variants of daughter 1, daughter 2, mother and father.
output: High-quality germline (Vg), candidate somatic variant set (Vs), and candidate de novo (Vn) mutation set. $\bar{v}_i^j$ represents the genotype of ith variant in sample j, $\bar{v}_i^j \in \{0,1,2\}$; $\bar{v}_i^j = 0$: no mutation, $\bar{v}_i^j = 1$: heterozygous mutation, $\bar{v}_i^j = 2$: Homozygous mutation.

```
procedure filterVariantsWithMendelianRule(V)
Set Vg = {}, Vs = {}, Vn = {}
for v_i in V do:
    if (v_i^d1[0] == v_i^d2[0] & v_i^d1[1] == v_i^d2[1])|(v_i^d1[0] == v_i^d2[1] & v_i^d1[1] == v_i^d2[0]):
        if ((v_i^d1[0] in v_i^f) & (v_i^d1[1] in v_i^m))|((v_i^d1[1] in v_i^f) & (v_i^d1[0] in v_i^m)):
            Vg.append(v_i)
        else:
            if (v̄_i^d1 == 1)& (v̄_i^m == 0)& (v̄_i^f == 0):
                Vn.append(v_i)
        end if
    else:
        if ((v̄_i^d1 == 1) |(v̄_i^d2 == 1))& (v̄_i^m == 0)& (v̄_i^f == 0):
            Vs.append(v_i)
    end if
end for
end procedure
```

4.2 Small variant detection

4.2.1 Small variant detection using Illumina reads

High quality SNVs and indels of Chinese Quartet were obtained from https://zenodo.org/record/5275189#.YaaYn9DMJPZ [11]. The variants of the four samples were normalized and merged using "norm" and "merge" command of bcftools (v1.13). We then phased the variants using whatshap [44] (v1.1). Variants violating the Mendelian rule were removed in the benchmarks.

4.2.2 Small variant detection using HiFi reads

HiFi reads were aligned to GRCh38 using minimap2 [60] (v2.20-r1061) with parameters "-a -H -k19 -O 5,56 -E 4,1 -A 2 -B 5 -z 400,50 -r 2000 -g 5000 --eqx --MD -Y " setting and then sorted using "sort" command of samtools [101] (v1.12). Subsequently, we called small variants for each sample using deepvariant [61] (v1.1.0) with parameter "--model_type=PACBIO" setting. gVCFs of four samples were merged and genotyped by glnexus (v1.2.7, https://github.com/dnanexus-rnd/GLnexus). SNVs and indels were phased according to their family information and HiFi reads using whatshap [44]. To obtain high-quality calls, we filtered variants to: (i) remove those with allele frequencies < 0.2, read depth < 25, read depth > 75 or length > 49bp; (ii) remove those violating the Mendelian rule, (discussed further in Supplementary Note), and (iii) retain only those where both twins had identical genotypes.

4.2.3 Small variant detection using haplotype-resolved assemblies

HiFi reads of the twin daughters were applied to hifiasm, hicanu, and flye. Then, we discovered variants with three haplotype-resolved assemblies using PAV [33] (v1.1.0) pipelines. Only variant supported by all three callsets was kept in following steps.

4.3 Structural variant detection

4.3.1 Structural variant detection by Illumina reads

Illumina reads of four samples were aligned to GRCh38 with bwa [76] (v0.7.17-r1188) "mem" command and the aligned reads were sorted by samtools [101] (v1.12). Next, PCR duplicated reads were marked by biobambam2 (v2.0.182). Then, structural variants were called using Manta [62] (v1.6.0), Delly [78] (v0.9.1), Lumpy [79](v0.2.13), and Pindel [80] (v0.3). More specifically, for Manta, we used default parameters for SV calling, retaining only those records which the caller flagged as "PASS". For Delly, we also used the "-x" parameter to exclude regions within the blacklist file provided by the HGSVC2 data portal. We ran Lumpy using Smoove (v0.2.8) with the '-x', '--genotype' and '--exclude' parameters, using the same blacklist file for the latter. For Pindel, we called variants using the parameters '-x2 -l -J' parameter, again providing the blacklist file for the latter. Finally, Pindel's output was converted to VCF format using pindel2vcf (v0.6.4) with parameters '-R GRCh38 -is 50 -as 100000000 -b -e 10 -ss 5'.

SVs shorter than 50bp were removed and only deletions (DELs), duplications (DUPs), insertions (INSs), and inversions (INVs) of canonical chromosomes were kept for following steps. Subsequently, four filtered callsets of each sample were integrated using Jasmine [81] (v1.1.5). Then, the merged SVs of four samples were compared and merged by Jasmine and only SV longer than 49bp were kept in final callset. Finally, we filtering the variants violating the Mendelian rule in benchmark callset.

For insertions/duplications merging, the Jasmine parameter was "-dup_to_ins --allow_intrasample --output_genotypes max_dup_length=1000000". For deletion merging, the Jasmine parameter was "--output_genotypes min_overlap=0.5 --allow_intrasample --output_genotypes".

4.3.2 Structural variant detection by HiFi reads

To obtain high-quality SV calls from HiFi reads of the Chinese Quartet, we utilized four popular callers, including pbsv (v2.6.2), Sniffles [39] (v1.0.12), CuteSV [40] (v1.0.11), and SVision (v1.3.6), to discover SV events and integrate their calls by Jasmine. For pbsv, we called SVs with "-t DEL,INS,INV,DUP,BND,CNV --ccs " setting. For Sniffles, we detected SVs with "–s 3". For cuteSV, we discovered SVs with "--max_cluster_bias_INS 1000 --diff_ratio_merging_INS 0.9 -s 2 --genotype --diff_ratio_merging_DEL 0.5 --max_cluster_bias_DEL 1000 --min_size 20 " setting. SVision calls were generated using the "--min_sv_size 30 -s10". For each caller, variants of four samples were filtered according to the read depth, allele depth, and Mendelian rule. Finally, only variants supported by at least two callers were kept in the following analysis. The SV merging process is same as the description in section 4.3.1.

4.3.3 Structural variant detection by haplotype-resolved assemblies

We assembled ONT reads using shasta and flye and HiFi reads using hifiasm, hicanu and flye, obtaining five haplotype-resolved assemblies for each haplotype. Then, we discovered structural variants with five haplotype-resolved assemblies using PAV pipelines. Only variant supported by at least three callsets was kept in following steps.

We used the PAV pipeline (https://github.com/EichlerLab/pav, v1.1.0) released by HGSVC to call variants from haplotype resolved assemblies. Notably, the PAV pipeline was developed with snakemake, and we directly used the default parameters to generate phased variants in this study. Then, the variants were split according to their variant types. For each type of SV (Deletion, Insertion, and Inversion), the variants from five assemblies were merged by Jasmine [81] with the --output_genotypes min_overlap=0.5 setting. The variants with at least three assemblies supported in Jasmine merged files were kept in the following analysis.

4.4 Complex variant and inversion detection

To discover the complex structural variants in our benchmark, we obtained complex variants from five callsets. HiFi reads were applied to pbsv (v2.6.2), Sniffles [39] (v1.0.12), CuteSV [40] (v1.0.11), and SVision (v1.3.6) [23], and SVs labeled as Inversion, CSV, and multiple types were extracted as candidates. The inversion of HRA calls were also extracted as candidates. For manual curation, the sequencing alignments of Illumina reads, HiFi reads, and HRAs in candidate regions were visualized by IGV [82]. The dotplot between HRAs and the reference genome of candidate regions was generated by Gepard [83]. We then manually inspected all dotplots and IGV snapshots associated with a reported CVS locus (Supplementary Table S10, Supplementary File 1). Dotplots were used to show the background of the reference genome in the region as well as the differences between our assemblies and the reference genome. IGV was used to display the alignment of Illumina reads, HiFi reads, and haplotype-resolved assemblies in the candidate variants.

4.5 Detection of *de novo* and putative somatic mutations

We utilized the Illumina and HiFi reads of four samples to detect *de novo* and somatic mutations in the twins. To obtain high-quality germline calls, de novo and putative somatic calls, the "Mendelian rule" filtering in this study included two aspects. First, variants with inconsistent genotypes in the monozygotic twin daughters were removing in the germline call set. The variants were also removed when the genotypes of the daughters contradicted those of their parents. To identify the de novo and somatic mutation, the variants shared by both twins are included in the candidate set for de novo mutation, while the variants specific to one twin daughter are included in the candidate set for putative somatic mutation. To further reduce the false positives, variants located in repeat regions, including STR, VNTR, SD, and SM, are excluded.

4.6 Benchmarking regions and high-confidence regions definition

We added “benchmark regions” and “high-confidence regions (Tier1)” for false positive identification. The "benchmark regions" covered all variants, while the "high-confidence regions" contained variants supported by at least two technologies or validated by an independent technology.

To define the benchmark regions, we first mapped the two haplotypes of assemblies to the GRCh38, and regions covered by both haplotypes were kept. The gaps regions of GRCh38, low confidence regions (LowConfidenceFilter.bed.gz, downloaded from HGSVC ftp), and regions with abnormal read depth (exclude.cnvnator_100bp.GRCh38.20170403.bed, downloaded from HGSVC ftp) were removed.

In this study design, HiFi reads and haplotype-resolved assemblies facilitated the detection of variants in complex regions, including simple repeats (SR), segmental duplications (SD), and variable number tandem repeats (VNTR), while short reads are not accessible well. Thus, variants in our benchmark were divided into Tier1 (high-confidence) and Tier 2 call sets according to whether they were in repeat regions and their supporting technologies.

For SVs, high-confidence calls (Tier 1) and corresponding regions of v2.1 of the benchmark were obtained according to the following steps:

a) Initially, we identified candidate high-confidence SVs within benchmark regions. This included all SVs supported by two or more technologies. For technology-specific SVs, we considered the following three criteria to classify them as candidate high-confidence SVs:

    i) They were not located within segmental duplications, VNTR (variable number tandem repeat), or simple repeat regions.

ii) They were not reported through short-read analysis.

iii) They were reported in an orthogonal callset.

b) Next, we refined the benchmark regions to create Tier 1 (v2.1) regions by excluding the following:

   i) Regions spanning SVs that did not meet the criteria in step a) were excluded.

   ii) Non-diploid regions were excluded.

   iii) Simple repeats that were overlap with variants not meet the criteria in step a) were excluded.

c) The candidate high-confidence SVs fully within the Tier 1 regions were defined as high-confidence SVs.

For small variants, high-confidence calls (Tier 1) of v2.1 were obtained according to the three steps:

a) Initially, we identified candidate high-confidence small variants within benchmark regions. This included all small variants supported by all three technologies. For variants supported by two technologies, we considered those were not located within segmental duplications, VNTR, simple repeat, or short tandem repeat regions as candidate high-confidence calls.

b) Next, we refined the benchmark regions to create Tier 1 (v2.1) regions for small variants by excluding the following:

   i) Regions spanning SVs were excluded.

   ii) Non-diploid regions were excluded.

   iii) Regions spanning small variants that did not meet the criteria in step a) were excluded.

iv) Repeat regions (Simple repeats, segmental duplications, short tandem repeats, and VNTRs) that were overlap with small variants not meet the criteria in step a) were excluded.

c) The candidate high-confidence small variants fully within the Tier 1 regions were defined as high-confidence small variants.

In our study design, users can benchmark their call set in custom regions and also in off-the-shelf regions. For example, users can compare variants in the relatively conservative Tier 1 regions or in more challenging repetitive regions. This customizable range of comparisons expands the utilities of the benchmark.

In the benchmarking set, to determine whether a variant belongs to a specific region, we require that the variant is 100% within that region. Users have the flexibility to adjust this parameter through the configuration file. In the query set, we also include variants in the bordering regions of a given region, such as Tier 1. This inclusion helps mitigate false negatives caused by inaccurate variant breakpoints. Users can manually verify the accuracy of variants in these border regions. When calculating false positives, variants in the bordering regions are excluded. The default size for the border regions is set at 100 bp for SV comparison, but users can also adjust this parameter.

Furthermore, to address the potential bias introduced by differences in variant lengths during comparisons, we have implemented a certain tolerance setting. For example, when comparing SVs with a length of 50 bp or greater, we include variants larger than 30 bp in both the initial query set and the benchmark set. However, when calculating True Positive, False Positive, and False Negative, we only consider variants that are 50 bp or longer. This setting ensures accurate identification of variants around the 50 bp threshold, preventing false positives and false negatives.

4.7 Phasing regions definition

To defined the phasing regions of the benchmark, we aligned the phased reads to the

reference genome. The regions with read depth more than 1/3 of the whole genome sequencing depth were extracted for each haplotype. We then merged the regions of two haplotypes and only regions supported by both haplotypes were included in final phasing regions.

5 Variant benchmark evaluation

In this study, we utilized the reads from Illumina, HiFi, and haplotype-resolved assemblies for variant discovery, while BGI and ONT reads were used as orthogonal technologies for providing additional evidence and determining whether technology-specific variants were included in Tier 1 or Tier 2. To obtain a high-quality variant benchmark with low false positives and high sensitivity, we applied strict filtering of the variant within each variant discovery technology and integrated the filtered variants generated by all three technologies (Illumina, HiFi, and haplotype-resolved assemblies). For variant validation, we used more flexible criteria to generate the initial BGI and ONT calls.

For BGI sequencing, we used deepvariant to call the variants and filtered variants with allele frequencies < 0.2. The variants were then normalized using the "norm" command in bcftools.

For ONT sequencing, we used pbsv (v2.6.2), Sniffles (v1.0.12), CuteSV (v1.0.11), and SVision (v1.3.6) to call SVs and integrate the variants for different callers by Jasmine. For each caller, we removed variants with support read < 15. And only variants supported by at least two callers were kept.

To assess the validated rate of each variant type in our benchmarks, we calculated the validated rate of each variant type in our benchmarks by equation (1-5).

$$P^{m,v} = \frac{N^{m,v}}{N^{m}} \quad (1)$$

$$P_t^{m,v} = \frac{N_t^{m,v}}{N^{m,v}} \quad (2)$$

$$P_t^{m,s} = \frac{N_t^{m,s}}{N^{m,s}} \tag{3}$$

$$N^{m,s} = N_{ILM}^{m,s} + N_{HiFi}^{m,s} + N_{HRA}^{m,s} \tag{4}$$

$$N^{m,v} = N^{m} - N^{m,s} \tag{5}$$

Where $m \in \{SNV,\ Indel,\ Deletion,\ Insertion\}$ and $t \in \{ILM,\ HiFi,\ HRA\}$. $P^{m,v}$ was the validated rate of m variant type (Fig. 2d). N^{m} and $N^{m,v}$ were total count and validated count of variant type m, respectively. $N_t^{m,v}$ and $N_t^{m,s}$ denoted the number of validated and singleton variant detected by technology t. $N^{m,s}$ represented the total singleton variants number in our benchmarks. $P_t^{m,v}$ (x-axis of Fig. 2e) was used to denote the ability of technology t to detect validated variants. And $P_t^{m,s}$ (y-axis of Fig. 2e) was the ability of technology t to detect specific variants.
